# Nonaqueous fractionation and overexpression of fluorescent‐tagged enzymes reveals the subcellular sites of L‐theanine biosynthesis in tea

**DOI:** 10.1111/pbi.13445

**Published:** 2020-07-24

**Authors:** Xiumin Fu, Yinyin Liao, Sihua Cheng, Xinlan Xu, Don Grierson, Ziyin Yang

**Affiliations:** ^1^ Key Laboratory of South China Agricultural Plant Molecular Analysis and Genetic Improvement & Guangdong Provincial Key Laboratory of Applied Botany South China Botanical Garden Chinese Academy of Sciences Guangzhou China; ^2^ University of Chinese Academy of Sciences Beijing China; ^3^ Plant and Crop Sciences School of Biosciences University of Nottingham Loughborough UK; ^4^ Center of Economic Botany Core Botanical Gardens Chinese Academy of Sciences Guangzhou China

**Keywords:** amino acid, biosynthesis, *Camellia sinensis*, ethylamine, nonaqueous fractionation, subcellular localization, tea, l‐theanine

## Abstract

l‐Theanine is a specialized metabolite in the tea (*Camellia sinensis*) plant which can constitute over 50% of the total amino acids. This makes an important contribution to tea functionality and quality, but the subcellular location and mechanism of biosynthesis of l‐theanine are unclear. Here, we identified five distinct genes potentially capable of synthesizing l‐theanine in tea. Using a nonaqueous fractionation method, we determined the subcellular distribution of l‐theanine in tea shoots and roots and used transient expression in *Nicotiana* or *Arabidopsis* to investigate *in vivo* functions of l‐theanine synthetase and also to determine the subcellular localization of fluorescent‐tagged proteins by confocal laser scanning microscopy. In tea root tissue, the cytosol was the main site of l‐theanine biosynthesis, and cytosol‐located CsTSI was the key l‐theanine synthase. In tea shoot tissue, l‐theanine biosynthesis occurred mainly in the cytosol and chloroplasts and CsGS1.1 and CsGS2 were most likely the key l‐theanine synthases. In addition, l‐theanine content and distribution were affected by light in leaf tissue. These results enhance our knowledge of biochemistry and molecular biology of the biosynthesis of functional tea compounds.

## Introduction

Tea (*Camellia sinensis*) is an important economic crop that is widely distributed in more than 30 countries worldwide with the main centres of production in China, Kenya, India and Japan. Tea is the second most popular beverage globally, after water, owing to its health effects and characteristic flavour. Specialized metabolites in tea leaves, such as l‐theanine, catechins, caffeine and aroma compounds, are responsible for the representative character of tea and consumer preference (Fu *et al*., 2015; Ho *et al*., 2015; Wei *et al*., 2018; Yang *et al*., 2013; Zhou *et al*., 2020). Studying the biosynthesis of these characteristic specialized metabolites in tea plants provides essential information and technology to improve the value of tea plants and their products.

l‐Theanine (γ‐glutamylethylamine) is a representative metabolite compound in tea, occupying over 50% of the total amino acids. The structure of l‐theanine is similar to that of l‐glutamine (Gln), and it has similar properties. l‐Theanine formation involves the conversion of toxic ethylamine into the nontoxic amino acid form (Sasaoka *et al*., 1965). In addition, l‐theanine also plays an important role in the first step of nitrogen assimilation to l‐glutamine in tea (Oh *et al*., 2008). Studies have also indicated that l‐theanine serves as the first step in the synthesis of skeletal carbon compounds (Feldheim *et al*., 1986) and precursor of polyphenols during black tea production (Tanaka *et al*., 2005). l‐Theanine also plays important roles in tea product quality and function, contributing to the tea umami taste, and counteracts bitter and astringent tastes in tea infusions (Feng *et al*., 2014; Yamaguchi and Ninomiya, 2000). Furthermore, l‐theanine has beneficial functions for human health, including nerve‐related effects, relaxation, neuroprotection and enhancing concentration and learning (Egashira *et al*., 2007; Haskell *et al*., 2008; Lu *et al*., 2004).

Owing to its significant contribution to quality and function of tea, the distribution and biosynthesis of l‐theanine in plants have been the subject of extensive research. l‐Theanine is mainly distributed among various *Camellia* species and is particularly abundant in the tea plant (*C. sinensis*; Deng *et al*., 2010; Nagata and Sakai, 1984), where l‐theanine is biosynthesized from l‐glutamic acid and ethylamine under the action of l‐theanine synthetase (TS, EC 6.3.1.6) (Sasaoka *et al*., 1965). Genes *TS1* (DD410896) and *TS2* (DD410895) from tea were originally reported to synthesize l‐theanine *in vitro* (Okada *et al*., 2006). Recently, an additional root‐specific‐expressed *TSI* (gene ID: TEA015198.1) was found in the tea plant. *TSI* belongs to the plant GSΙ type enzyme group, showing similarities to the FluG protein in fungi, and it has been confirmed as catalysing production of l‐theanine biosynthesis in tea root tissue (Wei *et al*., 2018). All the known l‐theanine synthetase‐related genes showed highly homology to Gln synthetase (GS, EC 6.3.1.2) (Cheng *et al*., 2017). There are other *GS* homologous genes in tea plants which have not yet been tested for l‐theanine synthetase activity, and the roles of specific genes in the synthesis of l‐theanine in different tissues of the tea plant are also unclear.

l‐Theanine is distributed among nearly all tea seedling tissues but varies in concentration between different tissues, accumulating mainly in shoot and root tissues and is especially high in root tissue (Tsushida and Takeo, 1984; Wei *et al*., 2018). In addition, l‐theanine accumulation in tea leaves is affected by several factors such as light and salt, and darkness can increase l‐theanine content (Deng *et al*., 2013; Deng *et al*., 2012; Kito *et al*., 1968). Long‐term shade treatment has always been applied during tea plant growth to increase l‐theanine content in tea shoots (Yang *et al*., 2012). Despite this physiological understanding, however, there has only been limited functional characterization of genes involved in the biosynthesis of L‐theanine in tea and there is even less information available concerning the cellular or subcellular locations of the biosynthetic reactions, which is important for understanding the regulation of its biosynthesis.

In the present study, all *GS*‐related genes from tea plants were functionally characterized and the tissue expression patterns and subcellular localizations of their encoded enzymes were also investigated. A nonaqueous fractionation method was employed to determine the subcellular distributions of l‐theanine in different tea tissues and study the effect of light upon subcellular distributions of l‐theanine in tea shoots (containing one bud, stem and two leaves; Figure S1). Both enzyme and metabolite subcellular localizations were analysed together to determine the subcellular site of *in vivo* l‐theanine biosynthesis in tea plants. We aimed to answer two questions here: Do the sites of l‐theanine biosynthesis vary in different tissues? and Do the sites of l‐theanine biosynthesis change along with external environmental factors? This research contributes new unambiguous information about the biosynthesis of well‐known functional compounds obtained from the direct investigation of tea plants and also provides a good reference for research into metabolites in nonmodel plants without a genetic transformation system.

## Results

### **Subcellular distributions of** l‐**theanine in tea shoot and root tissues**

Accumulation patterns of l‐theanine and its key precursors l‐glutamate and ethylamine in the tea plant tissues were analysed by UPLC‐QTOF‐MS and GC–MS (Table S1). l‐Theanine, ethylamine and l‐glutamate were present in young leaves, stems, flowers and roots of the tea plant. l‐Theanine was mainly accumulated in the root and young leaf tissues, followed by the stem and flower tissues. The largest amount of l‐glutamate was found in the flower tissue, while the lowest level was found in the root tissue of tea plants. Ethylamine was mainly accumulated in the root tissues and showed a strong positive relationship with the l‐theanine content in these tissues.

**Figure 1:**
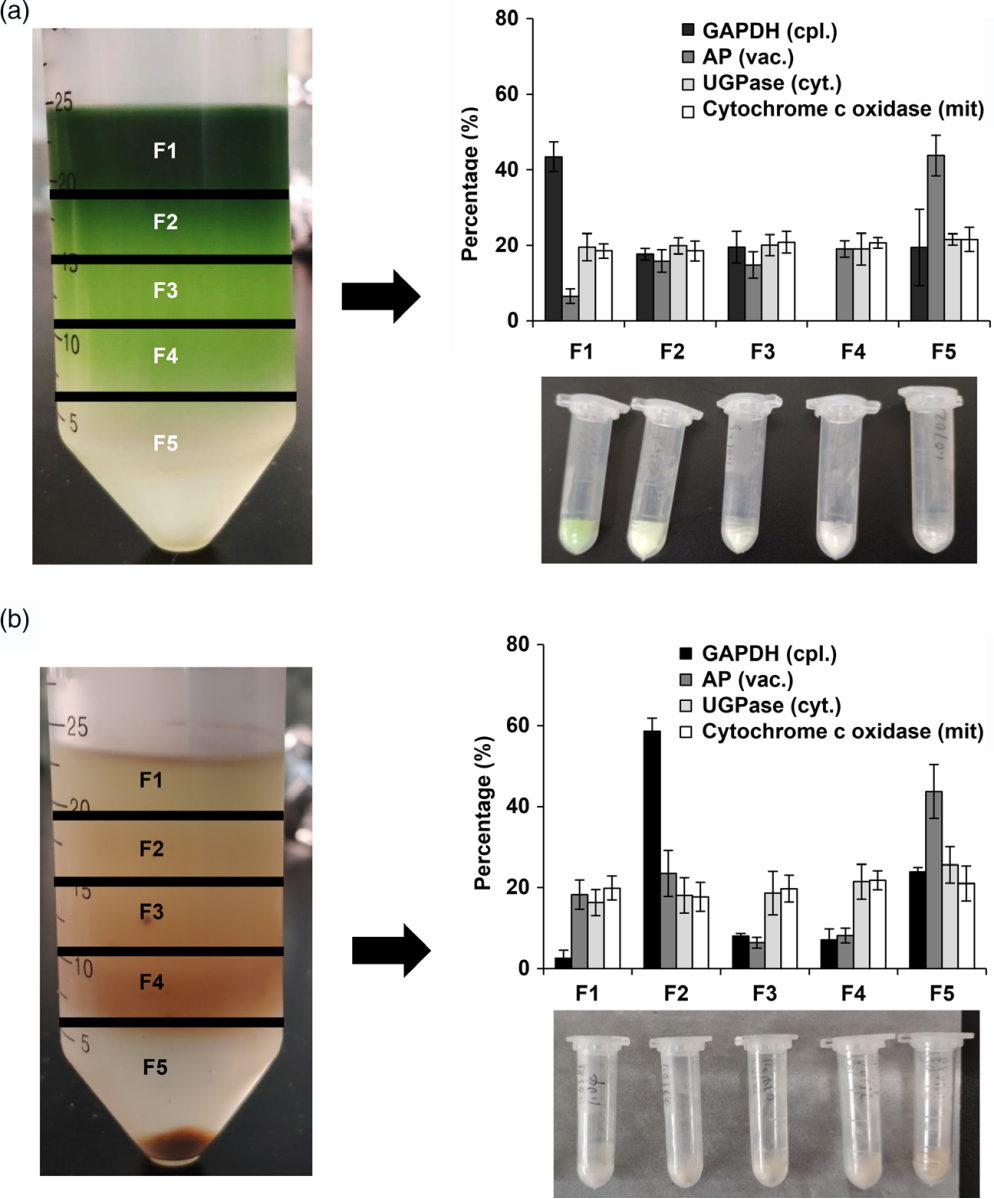
Distributions of compartment‐specific markers in nonaqueous gradients made using (a) shoot samples and (b) root tissues of tea. The tissues of tea shoot and root were collected from twenty‐year‐old tea plants (cv. Jinxuan) in March 2019. Tissues were fractionated using a nonaqueous fractionation procedure and the activities or contents of marker enzymes in the five fractions were determined. The distributions of plastidic (GAPDH), vacuolar (AP), cytosolic (UGPase) and mitochondrial (Cytochrome C oxidase) markers are shown as averages of three independent gradients. Data are expressed as percentage contents or activities in each fraction.

To further analyse the subcellular distribution of l‐theanine in tea plants, a nonaqueous density fractionation method was selected that has been used successfully to investigate subcellular metabolites in leaves (Stitt *et al*., 1989) and is applicable to the fractionation of tea shoot and root tissues (sampled in March, 2019) (Figure S2). The density was optimized to achieve good separation of the fractions obtained from the tea plant shoot and root tissues, with density ranges of 1.30–1.50 g/mL for tea shoot components and 1.30–1.55 g/mL for tea root components (Figure S2). Five fractions were obtained from the whole gradient (Figure 1). Compartment‐specific markers were determined in each fraction and expressed as a percentage of the total from the summed fractions of gradients for tea shoot or root tissues (Figure 1a and b) For the shoot material, a dark green ring was observed at the top of the gradient, corresponding to the chloroplast‐rich portion, accounting for 43.45% of the total, as indicated by the distribution of GAPDH as a marker. Acidic phosphatase (AP), as a vacuolar marker, showed clear enrichment, accounting for 43.82% of the total in the densest fraction (F5). Markers for the cytosol UGPase were evenly distributed throughout the gradient. The distribution of cytochrome c oxidase, a marker enzyme for mitochondria, resembled the cytosolic gradient profile (Figure 1). The distribution of marker enzyme in the root was similar to that in the shoot, except that the plastids showed an enrichment fraction in F2 accounting for 58.60% of the total. The marker for the vacuolar acidic phosphatase was also found to be concentrated in the heaviest fraction (F5), with 43.71% of the total in the root materials. UGPase for the cytosol and cytochrome c oxidase for the mitochondria were evenly distributed throughout the gradient in the root materials, similar to the patterns in the shoot tissue.

The amounts of l‐theanine and l‐glutamine in each fraction from the shoot and root tissues were detected by UPLC‐QTOF‐MS. Table 1 shows the percentage distribution of l‐theanine and l‐glutamine among different subcellular compartments in tea shoot or root tissues, as calculated and evaluated using BestFit (version 1.2) software (Klie *et al*., 2011). The l‐theanine content located within the chloroplast was 30.67%, with the rest (69.33%) found in the cytosol in the tea shoot tissue. The distribution of l‐theanine in tea shoot cells was similar to the l‐glutamine distribution pattern (35.00% located within the chloroplast; 65.00% located within the cytosol). However, l‐theanine was almost exclusively distributed in the cytosol in the root tissue, accounting for 99.00% of the total content.

**Table 1:** Subcellular distributions of L‐theanine and L‐glutamine in tea shoot and root tissues

Tissue	Metabolite	% of total tissue content			
		Plastid/Chloroplast	Vacuole	Cytosol	Mitochondria
Tea shoot	L‐Theanine	30.67 ± 15.59	n.d.	69.33 ± 15.59	n.d.
	L‐Glutamine	35.00 ± 14.44	n.d.	65.00 ± 14.44.	n.d.
Root	L‐Theanine	1.00 ± 1.41	n.d.	99.00 ± 1.41	n.d.
	L‐Glutamine	7.00 ± 2.83	n.d.	88.50 ± 3.54	4.50 ± 6.36

The tissues of tea shoot and root fractionated using a nonaqueous procedure were collected from twenty‐year‐old tea plants (cv. Jinxuan) in March 2019. L‐Theanine and L‐glutamine in each fraction were measured by UPLC‐QTOF‐MS. The subcellular distributions were calculated by comparing the metabolite and marker enzyme distributions using Bestfit software. The results represent the means ± S.D. of measurement on five different fractionations with three replicates. n.d., not detected.

The tea shoot sampled in November 2019 (Table 2) showed a similar l‐theanine distribution pattern to that in March 2019 (Table 1). However, l‐theanine was detected in the vacuole fraction (27.00%) in addition to the chloroplast (27.33%) and cytosol (45.67%) fractions of the tea shoots sampled in May 2020 (Table 2). The distribution of l‐theanine in tea shoots cells is likely to be a dynamic process, varying among subcellular organs during different seasons. The chloroplast and cytosol are demonstrated to be the two main locations for l‐theanine accumulation (Tables 1 and 2).

**Table 2:** Effect of shading treatment on subcellular distributions of L‐theanine and L‐glutamine in tea shoots

Sampling time			% of total tissue content			
	Metabolite	Treatment	Chloroplast	Vacuole	Cytosol	Mitochondria
November 2019	L‐Theanine	Nature	25.50 ± 2.12	n.d.	74.50 ± 2.12	n.d.
		Shade	n.d.	20.00 ± 7.07	80.00 ± 7.07	n.d.
	L‐Glutamine	Nature	41.00 ± 9.90	n.d.	59.00 ± 9.90	n.d.
		Shade	3.00 ± 2.83	n.d.	97.00 ± 2.83	n.d.
May 2020	L‐Theanine	Nature	27.33 ± 8.96	27.00 ± 8.19	45.67 ± 5.77	n.d.
		Shade	n.d.	n.d.	64.00 ± 15.87	36.00 ± 15.87
	L‐Glutamine	Nature	41.33 ± 3.51	24.00 ± 5.57	24.67 ± 2.08	n.d.
		Shade	4.67 ± 5.72	n.d.	93.67 ± 7.76	1.67 ± 2.04

Tea shoots from twenty‐year‐old tea plants (cv. Jinxuan) sampled at two different times (The duration of the experiment was 2 weeks from the 5 November 2019 to the 19 November 2019; the 24 April 2020 to the 8 May 2020) were applied to study the effect of shade treatment on the subcellular distributions of L‐theanine and L‐glutamine. n.d., not detected.

### CsGS/TSs involved in the biosynthesis of l‐theanine in tea shoot and root tissues

Three genes in tea plants, namely, *TS1* (DD410896), *TS2* (DD410895) and *TSΙ* (Gene ID: TEA015198.1), encoded proteins with the ability to synthesize l‐theanine *in vitro* and *in vivo* (Okada *et al*., 2006; Wei *et al*., 2018). Of these, *TS1* and *TS2* gene sequences in Okada *et al*. (2006) showed high homology with the *Arabidopsis GS1* (glutamine synthetase) gene, and the *TSΙ* gene sequence in Wei *et al*. (2018) showed high homology with the fluG gene of *Vitis vinifera*. Furthermore, two additional l‐glutamine synthetases (GSII1.1 and GSII2) genes were found in the tea genome database (Wei *et al*., 2018), which also showed a highly similar sequence homology to *Arabidopsis GSs*. These glutamine synthetase homologous genes *TS1* in Okada *et al*. (2006), *TS2* in Okada *et al*. (2006), *GSⅡ1.1* and *GSⅡ2* are denoted as *CsGS1.1*, *CsGS1.2*, *CsGS1.3* and *CsGS2*, respectively, in this study, and the *fulG* gene with homology to *TSΙ* in Wei *et al*. (2018) is denoted as *CsTSΙ*.

**Figure 2:**
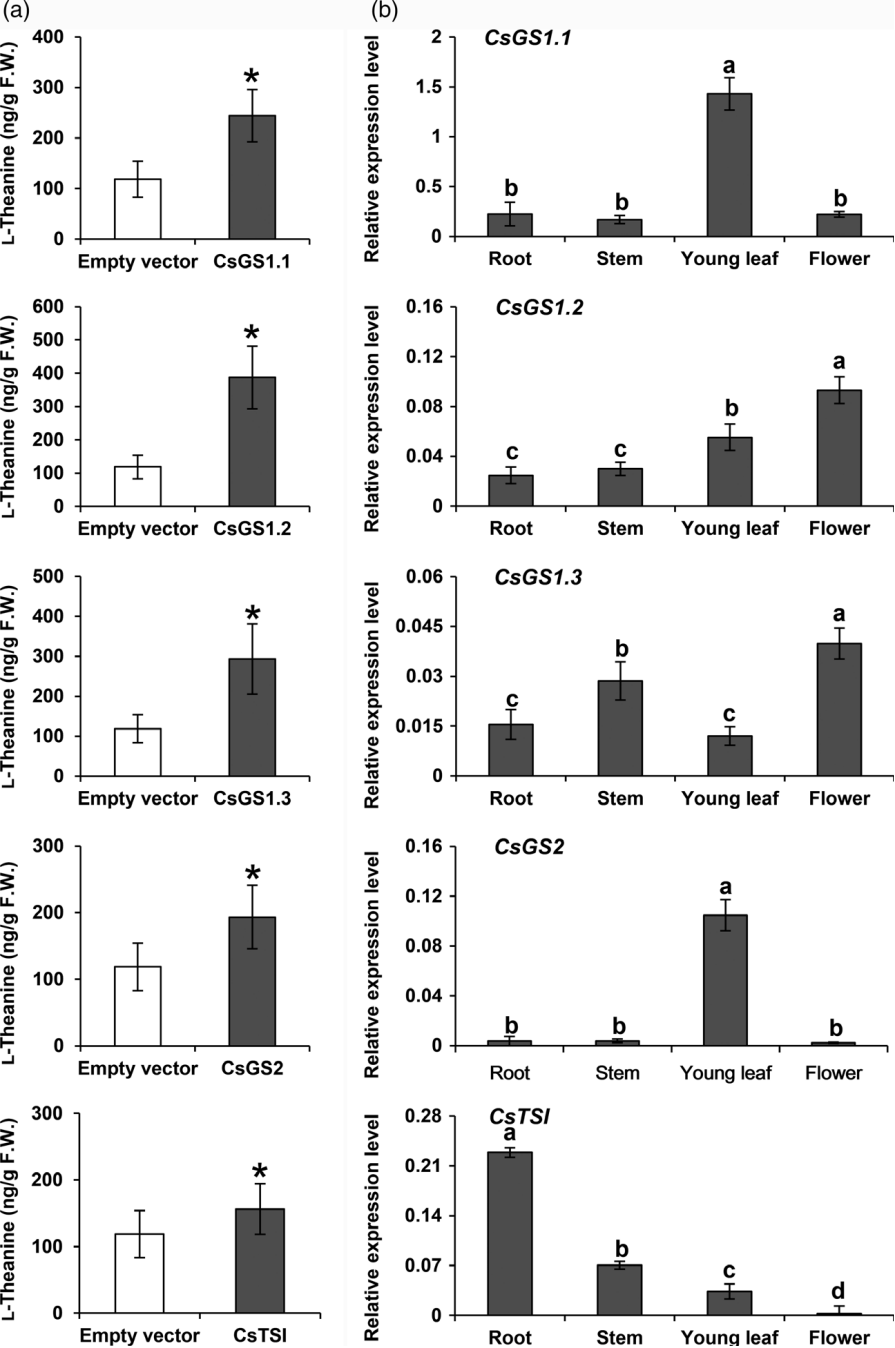
Analysis of the functions of CsGS/TSs and their expressions in tea plants. (a) Analysis of recombinant CsGS/TSs enzyme activities overexpressed in *N. benthamiana*. The tea *CsGS*/*TSs* gene was cloned into a pCAMBIA3300 vector and transiently overexpressed in *N. benthamiana*, driven by the 35S promoter. Overexpressing leaves were fed with ethylamine chloride solution (2 mg) for 24 h. L‐Theanine synthesized by the leaves was extracted and measured. Data are expressed as means ± SD (*n* = 4 or 5). * *P* ≤ 0.05 using Student’s *t* test. (b) mRNA levels of *CsGS1.1*, *CsGS1.2*, *CsGS1.3*, *CsGS2* and *CsTSΙ* in different tissues of tea plants. Tissues of Jinxuan cultivar (flower, young leaf, stem and root) used to study gene expression patterns were collected from the 4‐year‐old tea seedlings in November 2019. Data show mRNA levels relative to actin mRNA and are expressed as means ± SD (*n* = 3 or 4). Different letters above bars indicate significant differences using Duncan’s tests (*P* ≤ 0.05).

To investigate the l‐theanine synthetase activity of these *CsGS*/*TSs*, transient *CsGS*/*TSs* overexpression in *N. benthamiana* plants was carried out. Feeding an ethylamine solution (1 mL per leaf, 2 mg/mL) into leaves separately overexpressing *CsGS1.1*, *CsGS1.2*, *CsGS1.3*, *CsGS2* and *CsTSΙ* in *N. benthamiana* resulted in significantly higher l‐theanine contents compared with the control (empty vector expressed in *N. benthamiana*; Figure 2a). These results showed that all l‐glutamine synthetase genes and *fulG* genes in tea plants have l‐theanine synthesis activity *in vivo*.

The different members of the l‐glutamine synthetase gene family of *Arabidopsis* show differential expression patterns in leaves, roots and seeds (Peterman and Goodman, 1991). The transcripts of l‐glutamine synthetase homologous genes in tea plants might also be expected to vary in the root, stem, young leaf and flower tissues, and thus, different enzymes might be responsible for l‐theanine production in these different tissues, because the enzymes encoded by these gene members all have l‐theanine synthase activities. To test this hypothesis, *CsGS1.1*, *CsGS1.2*, *CsGS1.3*, *CsGS2* and *CsTSΙ* transcript levels were measured in different tea tissues, including the root, stem, leaf and flower, by quantitative qRT‐PCR with their gene‐specific primers (Figure 2b). *CsGS1.1* and *CsGS2* mRNAs accumulated to high levels in green tissue, with the highest transcript levels found in the leaf tissue, and *CsGS2* transcripts were present at very low levels in all tissues, except the leaf tissue. The *CsGS1.2* and *CsGS1.3* transcripts were expressed in all tissues examined and showed the highest content in flower tissue. The *CsTSΙ* transcripts were present at high levels specifically in the root tissue and were present at very low levels in other tissues. Among these genes, *CsGS1.3* mRNA abundance was 10‐ to 100‐fold less than that of other *GS* mRNAs in all tissues examined (Figure 2b). These results suggested that *CsGS1.1* and *CsGS2* were mainly responsible for l‐theanine biosynthesis in the leaf tissue, while *CsGS1.2* might be responsible for l‐theanine biosynthesis in the flower tissue, whereas *CsTSΙ* played a role in l‐theanine biosynthesis specifically in the root tissue, and *GS1.3* was unlikely to make a significant contribution because of its low abundance.

### Subcellular localizations of CsGSs/TS

To investigate which subcellular organelles are involved in the synthesis of l‐theanine in tea, the subcellular localizations of all CsGS/TSs enzymes were studied, because each of these proteins is likely to be capable of functioning in the synthesis of l‐theanine in addition to l‐glutamine in tea. After analysis by TargetP online using their predicted amino acid sequences, all the GS/TSs proteins (CsGS1.1, CsGS1.2, CsGS1.3 and CsTSΙ) except CsGS2 were predicted to have no N‐terminal presequences. CsGS2 was predicted to contain a chloroplast transit peptide, and a potential cleavage site was predicted after the first 50 amino acids. Alignment of amino acid sequences of these genes suggested that CsGS2 also had a potential transit peptide (Figure S3).

**Figure 3:**
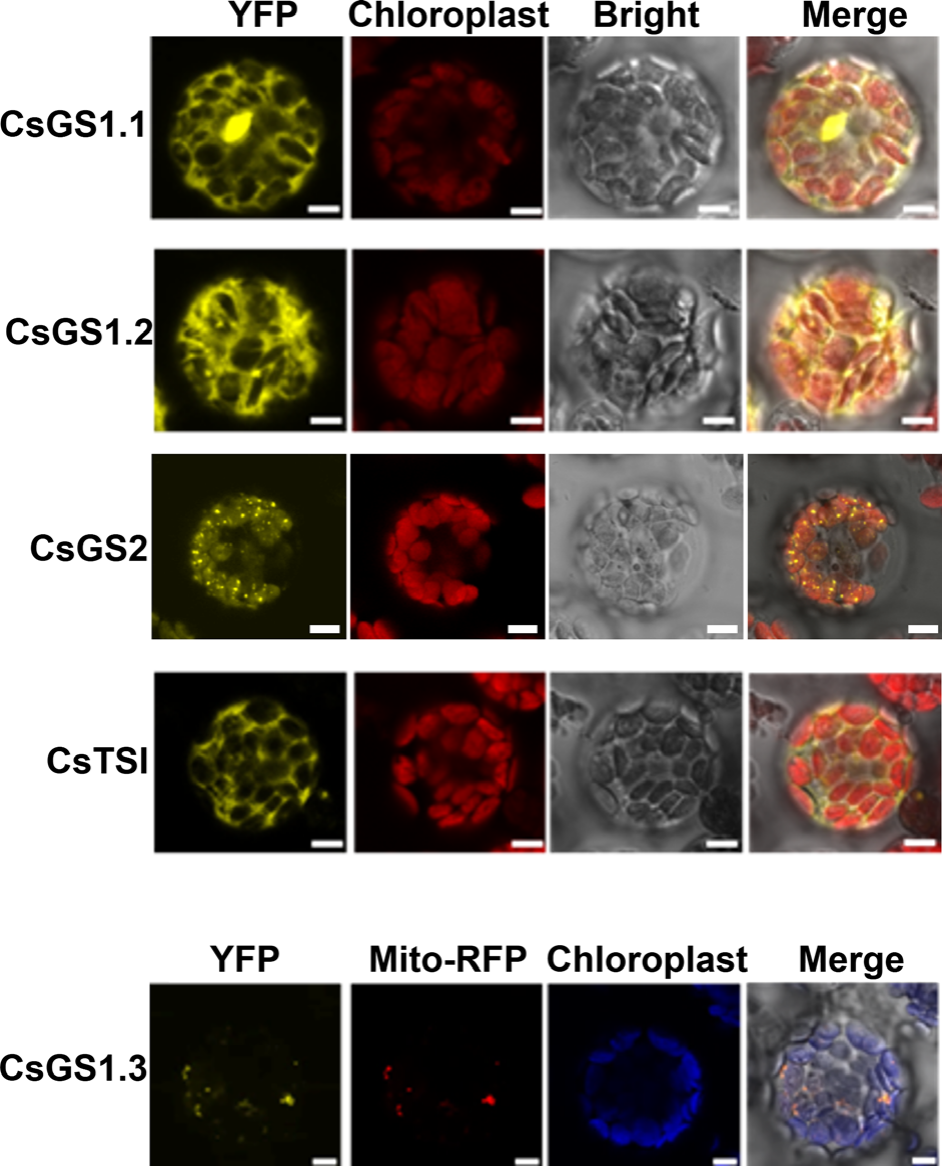
Subcellular localizations of CsGS/TSs in tea plants. The CsGS/TSs‐YFP fusion protein was transiently expressed in *Arabidopsis* protoplasts. Yellow fluorescence indicated CsGS/TSs‐YFP fusion protein Bar = 5 μm.

To identify the subcellular localizations of CsGSs, constructs of 35S:: CsGS1.1‐YFP, 35S:: CsGS1.2‐YFP, 35S:: CsGS1.3‐YFP, 35S:: CsGS2‐YFP and 35S:: CsTSΙ‐YFP encoding the CsGS1.1‐YFP, CsGS1.2‐YFP, CsGS1.3‐YFP, CsGS2‐YFP and CsTSΙ‐YFP fusion proteins, respectively, under the control of the 35SCaMV promoter were transformed into *Arabidopsis* protoplasts and the corresponding transient YFP‐tagged proteins were localized using confocal laser scanning microscopy (Figure 3). YFP signals were detected both in the cytosol and nucleus of protoplasts expressing CsGS1.1‐YFP (yellow). Protoplasts transformed with constructs 35S:: CsGS1.2‐YFP and 35S:: CsTSΙ‐YFP accumulated fluorescence in the cytosol. YFP fluorescence was observed in the chloroplasts and mitochondria of protoplasts expressing CsGS2‐YFP. In addition, the CsGS2‐YFP protein (without its predict N‐terminal chloroplast transit peptide) was found not to localize to chloroplast, but was, however, found in mitochondria (Figure S4). This suggests that in addition to the N‐terminal chloroplast targeting peptide in the CsGS2 gene product, a mitochondrial targeting peptide might occur after the chloroplast targeting peptide although this was not predicted by the computer program (the possible peptide is marked in grey in Figure S3). YFP fluorescence signals were also located in the mitochondria of protoplasts expressing CsGS1.3‐YFP (yellow), as confirmed by colocalization with the fluorescent signals of Mito‐RFP (a mitochondrial marker). In order to study whether a targeting signal exists at the C‐termini of these proteins, inverted constructs (YFP‐GS/TS) were also built and transformed into *Arabidopsis* protoplasts. It was found that all these proteins were localized in the cytosol, which suggested that there was no C‐terminal targeting sequence contained in these GSs/TS proteins (Figure S5).

As the transcripts of CsTSI were mainly detected in tea root tissue, transient *Agrobacterium*‐mediated gene expression in the *Arabidopsis* hydroponics root system was further applied to confirm the subcellular location of CsTSI. GFP signals were also detected in the cytosol of roots expressing CsTSΙ‐GFP (Figure S6), which confirmed that CsTSI was located in the cytosol of cell.

### Altered l‐theanine content in tea plants after shading treatment

**Figure 4:**
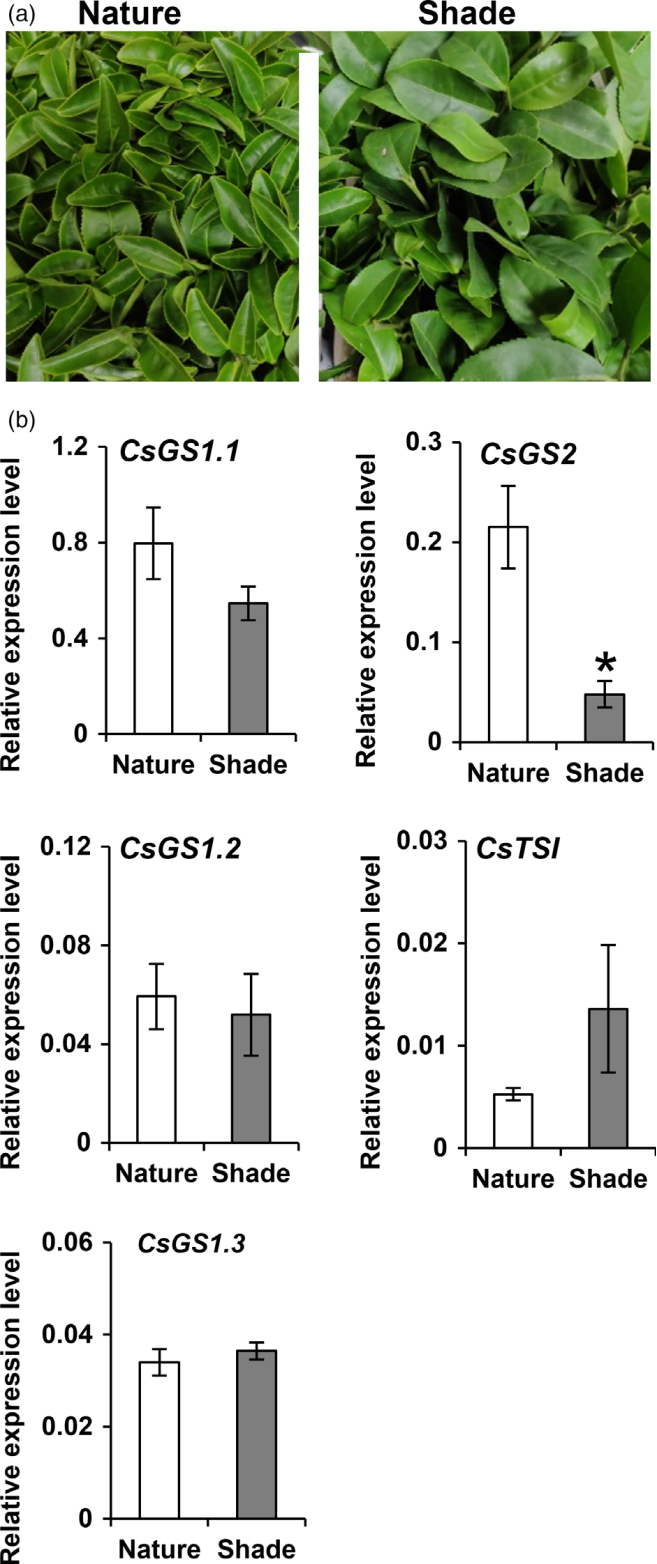
Effect of long ‐term shading treatment on *CsGS*/*TSs* expressions in tea shoots. (a) Photographs showing the twenty‐year‐old tea plant (cv. Jinxuan) grown in natural day/night cycles and long‐term shading environment for two weeks from 5 November 2019 to the 19 November 2019. (b) mRNA levels of *CsGS1.1*, *CsGS1.2*, *CsGS1.3*, *CsGS2* and *CsTSΙ* in the shoot of tea grown in natural light/dark cycles and long‐term shading environment for two weeks from 5 November 2019 to the 19 November 2019. * *P* ≤ 0.05 using Student’s *t* test.

l‐Theanine content increased in tea shoot after two‐week shading treatment in two different seasons (April–May 2020 and November 2019; Figures S7 and S8b). However, the long shade treatment also decreased the transcripts of *CsGS1.1* and *CsGS2*, with the leaf‐specific expression of *CsGS2* gene significantly down‐regulated in both seasons (Figure 4b and Figure S8c). In view of this, it was important to explain why the l‐theanine levels increased, while the transcripts of the two l‐theanine synthesis genes mainly expressed in leaves decreased after shading treatment. First, the content of l‐theanine synthesis substrate of l‐glutamate was measured after shading treatment and the levels showed an increase and a high correlation with the l‐theanine content (Figure S8b). Second, two‐year‐old tea seedlings were used to study whether other tissues, especially root tissue, are involved in the higher l‐theanine content in tea shoots after long‐term shade treatment (Figure S9). l‐Theanine significantly increased in root and stem tissue, but there was no effect on leaf tissue after two‐week shade treatment (Figure S10a). Furthermore, the substrate l‐glutamate only showed a slight increase in leaf tissues (Figure S10b). Another substrate, ethylamine, showed higher levels in root tissue after shading treatment of tea seedlings (Figure S10c), which might be related to the higher l‐theanine accumulation in root tissue. The patterns of gene expression of *CsGS1.1* and CsGS2 in tea leaves of tea seedlings after shade treatment (Figure S11) were similar to that of shoots of twenty‐year‐old tea plants (Figure 4b), with decreased transcript levels. The transcript level of the l‐theanine synthase gene *CsTSI* specifically expressed in root also showed reduction after long‐term shading (Figure S11). According to these results, we deduced that the higher l‐theanine content after shade treatment of tea shoot tissue could be partially explained because of the higher content of its substrate of l‐glutamate and also might be possibly related to the increased l‐theanine content in stems, where the increased of l‐theanine content may be due to translocation from the root tissue.

### Altered subcellular distribution of l‐theanine in tea shoots after shading treatment

It is known that long‐term shade can affect the l‐theanine content in tea shoots but whether it can lead to an alteration in the subcellular distributions in tea shoot is still unknown. Here, we analysed the subcellular distribution of l‐theanine in tea shoots after shade treatment (Figure 4a) and found that the subcellular distributions of metabolites in shaded tea shoot were quite different from those in naturally illuminated tea leaves (Table 2). l‐Theanine was mainly distributed in the cytosol (80.00%), followed by the vacuole, with very little in the plastids of tea shoot after long shade treatment in November 2019. About a quarter of l‐theanine was located within the chloroplasts of the naturally illuminated tea leaves (Table 2). Similarly, l‐theanine also showed a sharp decrease in chloroplasts in tea shoots after two‐week shade treatment from April to May 2020 (Table 2).

l‐Glutamine was almost exclusively distributed in the cytosol in the shaded tea leaves, accounting for 97.00% or 93.67% of the total content (Table 2), and very little was accumulated in chloroplasts. In naturally illuminated tea leaves, however, 41.00% or 41.33%% of the l‐glutamine content was located within the chloroplasts (Table 2). Thus, the shading treatment resulted in a dramatic decrease result in the distribution of both l‐theanine and l‐glutamine in chloroplasts, mainly because of the lower expression of *CsGS2* in tea shoot tissues.

## Discussion

### Use of nonaqueous fractionation procedure to study the subcellular distribution of metabolites in tea plants

In our previous study (Cheng *et al*., 2017), we have found that l‐theanine content differed between roots and leaves of the tea plant (*C. sinensis* var. Jinxuan), and in this study, we established that l‐theanine content varied among different tissues (Table S1). Several studies have suggested that l‐theanine is mainly synthesized in root tissue and then transported to young shoots (Dong *et al*., 2020; Sasaoka *et al*., 1965). Tea leaves, however, show l‐theanine synthesizing activity (Deng *et al*., 2009, 2010), although until now, it is still unclear whether l‐theanine biosynthesis occurs *via* the same route in root and leaf tissue.

Determining the subcellular localization of l‐theanine is an important step towards understanding the complexities of the enzymology and subcellular location of l‐theanine biosynthesis. Presently, knowledge of the locations of specialized metabolites involved in these pathways in tea plants is mainly inferred from localizations of the targeted enzymes involved following transient expression in model plants, such as *Arabidopsis* or *Nicotiana* (Gui *et al*., 2015; Zhou *et al*., 2017). Although observations with model plants can provide good reference information and might be used to answer scientific questions about economic crops, in recent years, increasing evidence has indicated that many differences exist between the biosynthesis of plant metabolites in model plants and crops. As a genetic transformation system for tea has yet to be established, obtaining direct *in vivo* evidence concerning the localization of enzymes involved in characteristic specialized metabolite biosynthesis in tea is difficult. An important approach to directly investigate metabolites at the cellular and subcellular levels in whole plant leaves or other tissues is to use a nonaqueous fractionation procedure. This method, which was improved by Gerhardt and Heldt (1984), prevents soluble compounds moving between compartments and different cell fractions after homogenization of the plant tissue.

**Figure 5:**
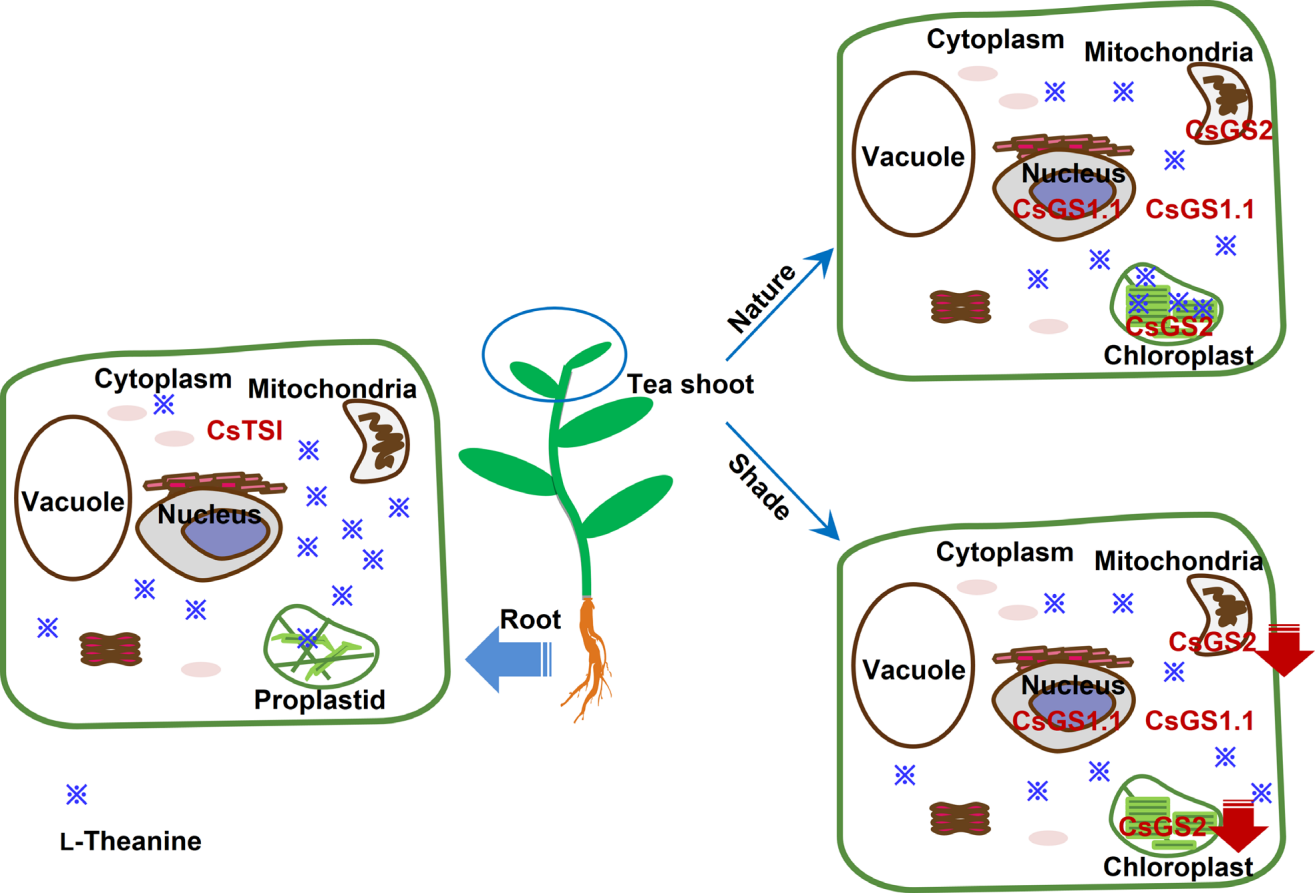
Schematic representation of the biosynthesis of L‐theanine in tea plants. L‐Theanine biosynthesis sites and accumulation pattern in root and shoot tissues are different. Further, L‐theanine distribution in tea shoot was affect by long‐term shading treatment. The model is that, in tea root tissue, L‐theanine was mostly distributed in the cytosol by possessed the root‐specific expression of cytosolic *CsTSI* gene; in tea shoot tissue, which grow in nature environment, L‐theanine was distributed mainly in the cytosol and chloroplasts by possessed the leaf‐specific expression of cytosolic *CsGS1.1* gene and plastidic *CsGS2* gene, respectively. In addition, if the tea shoot grows in long‐term shading environment, L‐theanine was distributed few in the chloroplasts mainly because of the declined expression of plastidic *CsGS2* gene. A red falling arrow indicates a decrease in gene expression. ※ distribution represents the distribution of L‐theanine content in cells.

Here, the subcellular distribution of specific l‐theanine and l‐glutamine metabolites in tea shoot and root tissues was successfully determined using a nonaqueous fractionation method after optimization of the density gradient separation (Figure 1 and Figure S2). This method has been successfully applied to analyse primary metabolism in potato tubers (Farré *et al*., 2001), water‐soluble heteroglycans in *Arabidopsis thaliana* leaves (Fettke *et al*., 2005), the role of raffinose in stabilizing photosystem II of *Arabidopsis thaliana* leaves (Knaupp *et al*., 2011) and reprogramming of metabolism during cold acclimation in *Arabidopsis thaliana* (Hoermiller *et al*., 2017). In this study, we found that the subcellular distribution of l‐theanine varies in different tissue and the subcellular distribution of l‐theanine in the tea shoot will change when the external environment changes, for example by extended shading treatment (Figure 5). Utilizing the nonaqueous approach is likely to be valuable in the study of the subcellular location of the specific metabolites in tea and other plants.

### All five CsGS/TS members have the catalytic ability to synthesize l‐theanine *in vivo* and have specific expression patterns and subcellular locations

In tea plants, l‐theanine is synthesized from ethylamine and l‐glutamic acid by l‐theanine synthetase (Sasaoka *et al*., 1965). It is important to know which factor (l‐theanine synthetase or ethylamine content) determines whether the tea plants have the ability to produce a large amount of l‐theanine, a property not possessed by most other plants. In the study by Wei *et al*. (2018), wild‐type *Arabidopsis* seedlings were shown to accumulate l‐theanine when fed with ethylamine. In addition, we found in our previous study that many plant tissues (leaf or root) such as *C. sinensis*, *C. japonica*, *Arabidopsis*, *Zea mays* and *Solanum Lycopersicum* all had the ability to synthesize l‐theanine by converting ethylamine and l‐glutamic acid into l‐theanine when fed with ethylamine (Cheng *et al*., 2017).

The reported l‐theanine synthases (*TS1* and *TS2*) gene sequence in tea plants (Okada *et al*., 2006) are highly similar to l‐glutamine synthase genes in other plants, and the *CsTSΙ* gene sequence in Wei *et al*. (2018) showed high homology to the nodulin gene or fluG gene in other plants (Cheng *et al*., 2017; Wei *et al*., 2018). Therefore, we expressed recombinant protein of l‐glutamine synthase genes of *Arabidopsis* (*AtGS1* and *AtGS2*) in our previous study and showed they have the ability to synthesize l‐theanine *in vitro* (Cheng *et al*., 2017). All these studies suggest that l‐theanine synthase in tea plants is actually l‐glutamine synthetase in other plants. Thus, tea plants can accumulate a large amount of l‐theanine just because they contain a large amount of ethylamine. In this study, we found that the l‐theanine content in different tissues of the same tea plant showed a good positive relationship with ethylamine content (Table S1). Therefore, the difference in l‐theanine accumulation between root and leaf is likely to be related to ethylamine content.

In this study, we verified the functions of the three previously reported genes and the two additional glutamine synthase homologous genes using transient expression in *Nicotiana*. All five genes tested had the catalytic ability to synthesize l‐theanine (Figure 2a). The question arises: Do these genes play a role in different tissues? We already know that different l‐glutamine synthetase gene family members fulfil the role in various tissues of many plants such as rice, *Arabidopsis* and maize (Guan *et al*., 2014; Hirel and Gadal, 1980; Moison *et al*., 2018; Swarbreck *et al*., 2010). As l‐theanine has a similar structure to l‐glutamine, the biosynthesis of l‐theanine might also occur *via* the same enzymes that are involved in l‐glutamine metabolism in plants.

Gene expression levels were analysed in different tissues (Figure 2b). The two genes *CsGS1.1* and *CsGS2* are preferentially expressed in green tissues. In addition, subcellular localization experiments revealed that CsGS1.1 is localized in the cytoplasm, and CsGS2 is localized in the plastids and possibly also the mitochondria (Figure 3). This suggested that l‐theanine may be distributed in these subcellular organelles in leaf tissue. *CsTSΙ* is expressed specifically in roots, which confirms the expression pattern shown by Wei *et al*. (2018). Further, *CsTSΙ* is localized in the cytoplasm (Figure 3; Figure S6), suggesting that l‐theanine may be distributed in the cytoplasm of root cells.

### l‐Theanine biosynthesis sites vary with tissues and external environmental factors

Through the subcellular localization of *CsGS*/*TSs* genes, we could predict the possible distribution of l‐theanine in cells of tea leaf and root tissues. In order to confirm the distribution of l‐theanine in these tissues directly, a nonaqueous method was applied in this study. The results indicated that, in tea shoot tissue, l‐theanine was mainly distributed in the chloroplasts and cytosol (Tables 1 and 2). However, in tea root tissue, l‐theanine was mostly distributed in the cytosol and absent from other subcellular organelles, including vacuoles and mitochondria (Table 1). These results were consistent with the subcellular localization of *CsGS*/*TSs* and tissue expression patterns (Figures 2 and 3).

Combining the subcellular locations of l‐theanine (Table 1) with the functional analysis (Figure 2a), expression patterns (Figure 2b) and subcellular locations of CsGS/TSs (Figure 3), we suggest that the cytosol in tea root cells is the main site of l‐theanine biosynthesis and that this occurs *via CsTSI*. This is consistent with previous conclusions from Wei *et al*. (2018) that the *CsTSI* gene plays an important role in the synthesis of l‐theanine in tea roots. The cytosol and chloroplasts, however, are the main subcellular locations in tea shoot cells that highly express both cytosolic *CsGS1.1* and chloroplast *CsGS2*, respectively, suggesting they play key roles in the synthesis and accumulation of l‐theanine in tea shoots. In addition, long shading treatment in tea plants could cause a decline in the ratio of l‐theanine in plastids through the decreased *CsGS2* expression level (Figure 4, Figure S8, and Figure S11). Light‐inducible plastidic GS2 has been confirmed in various plants, including barley, bean, pea and *Arabidopsis thaliana* (Freeman *et al*., 1990; Lightfoot *et al*., 1988; Peterman and Goodman, 1991; Tingey *et al*., 1988; Tjaden *et al*., 1995). In addition, CsGS2 in tea plant appears to be dual‐targeted to leaf chloroplasts and mitochondria, which showed the same subcellular location as l‐glutamine synthetase encoded by GLN2 in *Arabidopsis thaliana* (Taira *et al*., 2004).

Our study has determined the different *in vivo* subcellular sites of l‐theanine biosynthesis in tea shoot and root tissues using molecular and nonaqueous fractionation methods. The results indicate that under long‐term shade treatment compared to exposure to the natural day/night cycles, when the l‐theanine synthesis in chloroplasts is reduced, tea shoot tissue could derive more l‐theanine from tea roots, contributing to result in the changed distribution of l‐theanine in tea shoot. The strategies used in this study provide a good model for analysing the *in vivo* biosynthesis of specialized metabolites in economically important plants.

## Experimental procedures

### Plant materials

The tissues of tea (*C. sinensis* cv. Jinxuan) shoot containing parts of one bud, two leaves and the stem (Figure S1) and root used in the nonaqueous fractionation experiments were collected from twenty‐year‐old tea plants in March 2019. Other tissues of Jinxuan cultivar (flower, bud, leaf, stem and root) used to study gene expression patterns were collected from the 4‐year‐old seedlings in November 2019, in order to minimize the effects of any plant variation on the results. All the samples were collected from Yingde Tea Experimental Station of the Tea Research Institute, Guangdong Academy of Agricultural Sciences (Yingde, Guangdong, China). The tissue samples of Jinxuan cultivar for metabolites, molecular and nonaqueous fractionation analysis were immediately frozen in liquid nitrogen and stored at −80°C until further use.

### Preharvest shading treatment of tea plants

Tea shoot containing one bud, two leaves and the stem always were picked to produce the tea product. To study the light effect on l‐theanine content and subcellular distribution in tea shoot of tea plants, the samples were prepared as follows: twenty‐year‐old tea plants were used to study the l‐theanine content and subcellular distribution in tea shoots under shading stress conditions. A control group (Nature): full sunlight; shading treatment group: 30% full light. Throughout the experiment, neutral density black nylon net (six pin shade net, Sihui Huatong Metal Screen Products Co., Ltd., Guangdong, China) was used to reduce irradiance by 70% for the shading treatment. The duration of the experiment was two weeks in two different seasons from the 5 November 2019 to the 19 November 2019; from the 24 April 2020 to the 8 May 2020. After two weeks, tea shoots samples consisting of one bud, two leaves and the stem were picked and frozen in liquid nitrogen immediately and stored at −80°C until further use.

It is hard to study the transport and root affect the l‐theanine content in tea shoot of twenty‐year‐old tea plants. Two‐year‐old tea seedlings were used to study whether the root and transport ability participate in the l‐theanine accumulation in leaf under the same shading treatment (the duration of the experiment was two weeks from the 24 April 2020 to the 8 May 2020; Figure S9). Root, stem and leaf tissues of 2‐year‐old tea seedlings were collected (Figure S9b) and frozen in liquid nitrogen immediately and stored at −80°C until further use.

### Analysis of l‐theanine, l‐glutamate and l‐glutamine by UPLC‐QTOF‐MS

l‐Theanine, l‐glutamate and l‐glutamine were extracted and detected by UPLC‐QTOF‐MS. The extraction method and UPLC‐QTOF‐MS conditions for metabolite detection were taken from the study of Cheng *et al*. (2017), with details provided in the supplemental information.

### Ethylamine analysis by GC–MS

Internal ethylamine in tea samples was extracted according to the protocol of Tsushida and Takeo (1984). The detailed extraction method and GC‐MS conditions for metabolite detection were from Cheng *et al.,* (2017), with details provided in the supplemental information.

### Functional characterization of CsGS/TSs

The open reading frames (ORFs) of *CsGS1.1*, *CsGS1.2*, *CsGS1.3*, *CsGS2* and *CsTSΙ* were cloned from cDNAs prepared from tea tissues (Table S2). After sequencing confirmation, these ORFs were recombined into pCAMBIA3300 vector using an infusion system (In‐Fusion HD Cloning kits, Takara). The constructed and blank vectors were transformed into *Agrobacterium* GV3101 by electroporation. Overnight *Agrobacterium* cultures were centrifuged at 5000***g*** for 10 min, and the pellet was resuspended in a solution (10 mm MgCl_2_, 10 mm morpholineethanesulfonic acid, 100 μm acetosyringone, pH 5.6) to OD_600_ = 0.8. Leaves from *N. benthamiana* were infiltrated using a syringe without a needle. After 6 days, leaves from *N. benthamiana* were harvested and placed in a tube with distilled water containing ethylamine (2 mL, 1 mg/mL) with a blank as control. These tubes were incubated at 25°C for 1 day. The samples were then harvested and frozen in liquid N_2_ immediately, and the l‐theanine content was determined by UPLC‐QTOF‐MS, as described above.

### Transcript expression analysis of *CsGS*/*TSs*

Total RNA from tea tissue was extracted from frozen powder using a Quick RNA isolation Kit (Huayueyang Biotechnology (Beijing) Co., Ltd., Beijing, China). The isolated total RNA quality was verified by the A260/A280 ratio (ranging from 1.9 to 2.1) and gel electrophoresis. RNA (about 1 μg) was used to synthesize the first‐strand cDNA using a PrimeScript RT Reagent Kit with gDNA Eraser (Takara, Japan). Gene‐specific primers of *CsGS*/*TSs* were designed for qRT‐PCR analysis (Table S3). The qRT‐PCR analysis method is described in the supplemental information. Changes in the mRNA level of target genes for each treatment were normalized to that of *beta‐actin*.

### Transient expression of CsGS/TSs‐YFP fusion protein in *Arabidopsis* protoplasts

The protein sequences of CsGS/TSs were first aligned by ClustalX2, and potential localizations were predicted by TargetP (http://www.cbs.dtu.dk/services/TargetP/). Further, the ORFs of the five CsGS/TSs transcripts were subcloned into pSAT6‐EYFP‐N1 vector (linearized by restriction digest with BamHΙ and SalΙ) with specific primers by the infusion method without restriction site in final product (Table S4) according to the method of Zhou *et al*. (2017) and Takara online in‐fusion‐cloning‐tools (https://www.takarabio.com/learning‐centers/cloning/in‐fusion‐cloning‐tools). Inverted constructs (YFP‐CsGS/TSs) were also generated. The ORFs of the five CsGS/TSs transcripts with specific primers were subcloned into pSAT6‐EYFP‐N1 vector (linearized by PCR with specific primers in Table S5) using the infusion method. The detailed transient expression process is provided in the supplemental information. For colocalization studies, cultures containing the constructs Mito‐RFP for labelling of mitochondria were coinfiltrated into protoplasts. Fluorescence signals were detected using a Zeiss LSM 510 confocal laser scanning microscope (Carl Zeiss, Jena, Germany).

### Subcellular localization through transient agrobacterium‐mediated gene expression in the *Arabidopsis* hydroponics root system

Thirty days of *Arabidopsis thaliana* seedlings were placed to a nutrient solution (pH 7.0) containing 300 μm NH_4_Cl, 300 μm KNO_3_, 1 mm CaSO_4_, 0.5 mm KH_2_PO_4_, 0.75 mm MgSO_4_ and 0.06 g/L Fe‐EDTA (Levy *et al*., 2005). After one week, an A. tumefaciens culture (0.5 × MS medium, 5% sucrose and 0.05% Silwet L‐77) replaced nutrient solution. cDNA of *CsTS* was cloned into the pCAMBIA3300 vector with the GFP codon region, and empty pCAMBIA3300 vector was used as a control. Both vectors were transformed into *Agrobacterium* GV3101. Overnight *Agrobacterium* cultures were centrifuged at 5000***g*** for 10 min, and the pellet was resuspended in a solution (0.5 × MS medium, 5% sucrose and 0.05% Silwet L‐77) to a final absorbance at 600 nm of 1.0. The suspension was used to transform the roots of *Arabidopsis* plants grown in hydroponic culture. The *Arabidopsis* plant dipped in *Agrobacterium* cultures and was subjected to 20 min of vacuum. The roots of *Arabidopsis* plant were rinsed by water twice, then incubated in water containing with 250 mg/L cefotaxime for 30 min and then rinsed with water and back to the hydroponics nutrient solution. After 3 day, the roots were observed under a confocal microscope.

### Nonaqueous fractionation methods for tea shoot and root tissues

Nonaqueous fractionation of tea shoot and root tissues was performed according to a method reported by Stitt *et al*. (1989); Farré *et al*. (2001); Krueger *et al*. (2014), with slight modifications, as detailed in the supplemental information.

The protein contents of the marker enzymes (UDP‐Glucose‐Pyrophosphorylase, cytosol marker; AP, vacuole marker; Cytochrome c oxidase, mitochondrial marker) in different fractions were detected using their corresponding ELISA kits. The GAPDH (plastid marker) activity was measured from the decrease in NADPH concentration at 340 nm according to Krueger *et al*. (2014).

l‐Theanine and l‐glutamine from the dried samples were extracted with methanol/chloroform/water (1.4 mL; 1:2:1, *v*/*v*/*v*), followed by centrifugation (12 000***g***, 10 min). The upper phase was used for l‐theanine content analysis by UPLC‐QTOF‐MS, as described above. A three‐compartment calculation program (BestFit) was employed to calculate the distribution of subcellular metabolites (Krueger *et al*., 2011).

### Statistical analysis

Statistical analysis of the data for gene transcripts levels and metabolites was carried out using SPSS Statistics software (version 19, IBM Corporation) with one‐way analysis of variance (ANOVA) with Duncan’s significant difference (D). The *P* value was set to 0.05.

## Conflict of interest statement

The authors have declared no conflict of interest.

## Author contributions

Z.Y. proposed the project, conceived and designed the experiments. X.F contributed to the experiments of enzyme functional characterization, transcript expression analysis and nonaqueous fractionation. Y.L. and S.C. contributed to metabolite analysis. X.X. contributed to subcellular localization analysis. X.F. and Z.Y. analysed the results. X.F. and Z.Y. wrote the manuscript. D.G. gave the instructive comments and revised the manuscript. All authors reviewed the manuscript.

## Supporting information


**Supplementary Methods**
**Figure S1** The photo of tea shoot tissue.
**Figure S2** Optimization of the density for separation of the fractions in tea tissues using the nonaqueous fractionation method.
**Figure S3** CsGSs amino acid sequences alignment by Clustal.
**Figure S4** Subcellular localization for CsGS2‐YFP without its predict N‐terminal chloroplast transit peptide.
**Figure S5** Subcellular localizations of YFP‐CsGS/TS.
**Figure S6** *Arabidopsis* hydroponics root culture (a) and subcellular localizations for CsTSI‐GFP (b).
**Figure S7** Effect of shade treatment on L‐theanine content in tea shoot tissue.
**Figure S8** Effects of shade treatment on L‐theanine accumulation in tea shoot tissue.
**Figure S9** Two‐year‐old tea seedling used for studying L‐theanine accumulation mechanism under the shade treatment.
**Figure S10** Analyses of L‐theanine (a) and its precursors of L‐glutamate (b) and ethylamine (c) contents in two‐year‐old tea seedling after long‐term shading treatment.
**Figure S11** Analyses of mRNA levels of *CsGS1.1*, *CsGS1.2*, *CsGS1.3*, *CsGS2,* and *CsTSΙ*in the tissues of two‐year‐old tea seedling after long‐term shading treatment.
**Table S1** Contents of L‐theanine and its precursors in different tea tissues.
**Table S2** Primers for vectors for transient overexpression in tobacco.
**Table S3** Primers for qRT‐PCR analyses.
**Table S4** Primers for CsGS/TS‐YFP‐vectors in *Arabidopsis* protoplasts.
**Table S5** Primers for YFP‐CsGS/TS vectors in *Arabidopsis* protoplasts.
