# Supplementary material for: Nonaqueous fractionation and overexpression of fluorescent‐tagged enzymes reveals the subcellular sites of L‐theanine biosynthesis in tea
Source: Plant Biotechnol J. 2020 Jul 24;19(1):98–108. doi: 10.1111/pbi.13445 (PMC7769230; doi:10.1111/pbi.13445)
Supplement: Supplementary file 1 — Supplementary Methods Figure S1 The photo of tea shoot tissue. Figure S2 Optimization of the density for separation of the fractions in tea tissues using the nonaqueous fractionation method. Figure S3 CsGSs amino acid sequences alignment by Clustal. Figure S4 Subcellular localization for CsGS2‐YFP without its predict N‐terminal chloroplast transit peptide. Figure S5 Subcellular localizations of YFP‐CsGS/TS. Figure S6 Arabidopsis hydroponics root culture (a) and subcellular localizations for CsTSI‐GFP (b). Figure S7 Effect of shade treatment on L‐theanine content in tea shoot tissue. Figure S8 Effects of shade treatment on L‐theanine accumulation in tea shoot tissue. Figure S9 Two‐year‐old tea seedling used for studying L‐theanine accumulation mechanism under the shade treatment. Figure S10 Analyses of L‐theanine (a) and its precursors of L‐glutamate (b) and ethylamine (c) contents in two‐year‐old tea seedling after long‐term shading treatment. Figure S11 Analyses of mRNA levels of CsGS1.1, CsGS1.2, CsGS1.3, CsGS2, and CsTSΙin the tissues of two‐year‐old tea seedling after long‐term shading treatment. Table S1 Contents of L‐theanine and its precursors in different tea tissues. Table S2 Primers for vectors for transient overexpression in tobacco. Table S3 Primers for qRT‐PCR analyses. Table S4 Primers for CsGS/TS‐YFP‐vectors in Arabidopsis protoplasts. Table S5 Primers for YFP‐CsGS/TS vectors in Arabidopsis protoplasts. [file PBI-19-98-s001.docx]

**Title:** Nonaqueous fractionation and overexpression of fluorescent tagged enzymes reveals the subcellular sites of L-theanine biosynthesis in tea

Authors: Xiumin Fu ^1^, Yinyin Liao ^1, 2^, Sihua Cheng ^1, 2^, Xinlan Xu ^1^, Don Grierson ^3^ and Ziyin Yang ^1, 2, 4,^ *

**Institutional addresses:**

^1^ Key Laboratory of South China Agricultural Plant Molecular Analysis and Genetic Improvement & Guangdong Provincial Key Laboratory of Applied Botany, South China Botanical Garden, Chinese Academy of Sciences, Xingke Road 723, Tianhe District, Guangzhou 510650, China

^2^ University of Chinese Academy of Sciences, No.19A Yuquan Road, Beijing 100049, China

^3^ Plant and Crop Sciences, School of Biosciences, University of Nottingham, Sutton Bonington Campus, Loughborough, LE12 5RD, United Kingdom

^4^ Center of Economic Botany, Core Botanical Gardens, Chinese Academy of Sciences, No. 723 Xingke Road, Tianhe District, Guangzhou 510650, China

***Corresponding author:** zyyang@scbg.ac.cn; Tel.: +86-20-38072989

**Supplemental methods**

**Nonaqueous fractionation of** **tea shoot and root tissues**

Nonaqueous fractionation for tea leaf and root tissues was according to the methods of Stitt *et al*., (1989); Farré *et al*., (2001); Krueger *et al*., (2014) with slight modifications. About 4 g fresh weight of leaf or root samples were homogenized in a ball mill precooled with liquid nitrogen for 20 s at 28 Hz. After homogenization, the homogenized material was placed into the lyophilizer at 0.02 bar and -50^o^C for 3 days. The resulting dry powder was resuspended in a plastic tube with 20 mL of a tetrachlorethylene-heptane mixture (66:34 [v/v]; density = 1.3 g cm^-3^; the mixture was stored with 3 Å molecular sieve and ultrasonicated for a total of 2 min, with 6 cycles of 10 s pulses and 10 s breaks at 65% power. The sonicated suspension was filtered through nylon net with a pore size of 20 μm; the net was washed 3 times with 10 mL of heptane, and centrifuged for 10 min at 3,200 *g* and 4^o^C. After centrifugation of the suspension, the organic supernatant was discarded and the pellet was resuspended in 3 mL C_2_Cl_4_/C_7_H_16_ mixture 66:34 (v/v). 500 μL in 10×50 μL aliquots were withdrawn (for determination of enzyme activity and metabolites in the unfractionated material), and the remaining 2.5 mL of the suspension was loaded on the top of the gradient. A linear gradient (25 mL, for shoot tissue between 1.30 and 1.50 g cm^-3^, and for root tissue between 1.30-1.55 g cm^-3^) was made using a gradient former connected to a peristaltic pump. The gradients were centrifuged for 1 h at 3,800 *g* at 4^o^C. The fractions (F1 to F5, 4-6 mL for each fraction) were carefully removed from the top using pasteur pipettes into a clean 50 mL tube. Three volumes of C_7_H_16_ were added to each tube and the suspension was mixed well. The suspensions were centrifuged for 10 min at 3,200 *g* at 4^o^C in a centrifuge swing-out-rotor. The supernatants were discarded and the pellet was resuspended in 5 mL C_7_H_16_, and 10 aliquots of 500 μL of the suspension were transferred into 2 mL tubes. The samples were dried in tubes by N_2_ for 1 hour, and then extracted for assay of enzymes and metabolites.

Proteins from the dried samples were dissolved in buffer A (50 mM Hepes-NaOH pH 7.4; 5 mM MgCl_2_; 1 mM EDTA; 1 mM EGTA; 0.1 % Triton X-100; 10 % glycerol; 2 mM benzamidine; 2 mM aminocaproic acid; 1.5 mM PMSF; 1 g/L PVPP) to determine the activities of GAPDH, UGPase and Cytochrome C oxidase; proteins from the dried samples were extracted by buffer B (0.5 M sodium acetate pH 5.0, adjusted with glacial acid) for determining the contents of proteins of acidic phosphatase.

**Analysis of L-theanine, L-glutamate and L-glutamine by UPLC-QTOF-MS**

The detailed extraction method and UPLC-QTOF-MS conditions for detecting metabolites were as described by Cheng *et al*. (2017). L-Theanine, L-glutamate, and L-glutamine were extracted and detected by UPLC-QTOF-MS as follows: 100 mg powdered tea tissues (obtained by grinding plant material in liquid nitrogen with a mortar pestle) were placed in a 2 mL tube, and 0.35 mL of cold methanol (100%) was added, with vortexing for 2 min followed by ultrasonic extraction in ice cold water for 20 min. 0.7 mL of chloroform and 0.35 mL of cold water were then added and vortexed for 2 min, the samples were then centrifuged (12000 *g*, 10 min) to obtain phase separation. The resulting upper layer was used as the crude extract of amino acids. The samples were filtered through a 0.22 μm nylon membrane filter and analysed by the UPLC-QTOF-MS system consisting of an ACQUITY UPLC I-Class (Waters, Milford, MA, USA) and Xevo® GS2 QTOF (Waters, Milford, MA, USA) equipped with a HSS T3 (1.8 μm, 100 × 2.1 mm) column (Waters Corp, Milford, MA) at 40 °C. The mobile phase consisted of water as solvent A containing 0.1% formic acid and acetonitrile as solvent B containing 0.1% formic acid with a gradient programme as follows: 0-5 min, 0% B; 5-5.1 min, 0% B to 90% B; 5.1-10 min, 90% B; 10-10.1 min, 90% B to 0% B; 10.1-13 min, 0% B. Flow rate was set of 0.25 mL/min. The input temperature was set at 4 ℃ and the injected sample volume was 5 μL. The capillary and sampling cone voltages were 2 kV and 50 V, respectively, in positive ESI mode. The source temperature and desolvation temperatures were 100 °C and 350 °C, respectively. The cone gas flow and the desolvation gas flow were 50 L/h and 600 L/h, respectively. Data were collected for the mass range 50-1200 Da in MS^E^ mode and analyzed using MassLynxTM software (Waters Corporation). The characteristic ions for L-theanine, L-glutamate and L-glutamine are *m/z* 175.1083, 148.0610, 147.0770, respectively.

**Analysis of ethylamine by GC-MS**

The samples were ground in liquid nitrogen with a mortar and pestle and the powder transferred to a 15 mL tube and steamed for 1 min and then were immediately frozen in liquid nitrogen. The frozen samples were lyophilized and the powdered samples (100 mg) were transferred to a 2 mL tube and diluted with 0.5 mL of distilled water. The tube was heated in a water bath at 80^o^C for 5 minutes and then allowed to stand for 2 h at room temperature. The extract was then filtered and centrifuged at 3500 × *g* for 5 min. The analytical method for ethylamine determination was as described by Almeida *et al*. (2012) with a small modification. An aliquot of sample (1 mL) was transferred to a 4 mL silanized screw-capped glass vial containing 1 mL of toluene; 1 mL of 0.5 M phosphate buffer (pH 12.0) was added to make the mixture alkaline and then 5 μg of a deuterated internal standard (IS) [^2^H_5_]ethylamine:HCl (2 mg mL^−1^) was added, together with 25 μL of IBCF. The vial was shaken for 10 min at 250 rpm and centrifuged at 3500 × *g* for 5 min. The resultant toluene (upper) layer (200 μL) was transferred to a clean vial, and 200 μL of alkaline methanol, prepared by dissolving KOH in methanol until saturation, followed by filtration through a 0.45 μm filter, was added. The tube was shaken for another 5 min, then 600 μL of 5 M NaOH was added, and the mixture was shaken for a further 5 min. After the mixture was centrifuged, the toluene layer was used for measuring ethylamine content. The ICBF derivatizated products were analyzed by the GC−MS method.

GC separation was performed on GC-MS coupled with an HP-5 ms column (30 m × 0.25 mm × 0.25 μm, Agilent Technologies, California, USA). The injector temperature was 280^o^C, splitless mode was used, and helium was the carrier gas with a velocity of 1.0 mL/min. The temperature program had an initial temperature of 80°C for 3.0 min, followed by ramping at 10°C/min to 160°C, and then at 25°C/min to 280°C, which was then maintained for 13.3 min. MS was operated in selective ion monitoring mode (*m/z* 90 and 72 for IBCF-ethylamine derivative; *m/z* 95 and 77 for IBCF-[^2^H_5_]ethylamine derivative).

**Analysis of *CsGS/TSs transcript levels***

Total RNA was extracted from frozen tea tissues samples with a Quick RNA isolation Kit (Huayueyang Biotechnology (Beijing) Co., LTD., Beijing, China). The isolated total RNA quality was verified by A260/A280 ratio (ranging from 1.9 to 2.1) and gel electrophoresis. About 1 μg RNA was used to synthesize the first-strand cDNA using a PrimeScript RT Reagent Kit with gDNA Eraser (Takara, Japan). Gene-specific primers of *CsGS/TSs* for qRT-PCR analyses were designed (Table S3). The qRT-PCR analyses were performed on a Roche LightCycler 480 with the reactions in a total volume of 20 μL, including 2 μL of each primer (10 μM), 2 μL of cDNA, 10 μL of iTaq Universal SYBR Green Supermix (Bio-Rad, USA), and 6 μL of ddH_2_O. The qPCR system program was initiated with a preliminary step of 30 s at 95^o^C, followed by 45 cycles at 95^o^C for 5 s and 60^o^C for 1 min. A melting curve program (65-95^o^C with a heating rate of 0.1^o^C per second) was generated for each sample at the end of each run to check the purity of the amplified products. No-template controls for each primer pair were included in each run. The mRNA levels of target genes for each treatment were normalized to that of *beta-actin*.

**Transient expression of CsGS/TSs-YFP fusion protein in *Arabidopsis* protoplasts**

The protein sequences of CsGS/TSs were first aligned by ClustalX2 and their subcellular localizations were predicted by TargetP (<http://www.cbs.dtu.dk/services/TargetP/>). The ORFs of the five CsGS/TSs transcripts were subcloned into pSAT6-EYFP-N1 vector (linearized by restriction digest with BamHΙ and SalΙ) with specific primers using the infusion method without restriction site in the final product (Table S4) according to the method of Zhou et al. (2017) and Takara online in-fusion-cloning-tools (<https://www.takarabio.com/learning-centers/cloning/in-fusion-cloning-tools>), pSAT6-EYFP-N1 vector. The inverted constructs (YFP-CsGS/TSs) were also generated. The ORFs of the five CsGS/TSs transcripts with specific primers were subcloned into pSAT6-EYFP-N1 vector (linearized by PCR with specific primers shown in Supplementary Table S4) using the infusion method (Table S5). The lower epidermal surface cell layer was peeled away using adhesive tape. The peeled leaves were transferred to a Petri dish containing 10 mL of enzyme solution (1.5% cellulose R10, 0.4% macerozyme R10, 0.4 M mannitol, 20 mM KCl, 20 mM MES, and 10 mM CaCl_2_), and allowed to stand for 4 h while protoplasts were released into the solution. The protoplasts were centrifuged at 100 *g* for 2 min and washed with cold W5 solution (150 mM NaCl, 125 mM CaCl_2_, 5 mM KCl, and 20 mM MES, pH 5.7) twice. Finally, protoplasts were resuspended in an MMG solution (0.4 M mannitol, 15 mM MgCl_2_, and 4 mM MES, pH 5.7). 10 μL of PCsGS1.1, PCsGS1.2, PCsGS1.3, PCsGS2 and PCsTSΙ plasmid DNA, respectively, (1 μg/μL) and 110 μL of polyethylene glycol 4000/Ca (PEG/Ca) solution were added to 100 μL of protoplasts (about 2×10^4^ protoplasts). After incubation for 15 min at room temperature, the mixture was diluted with 440 μL of W5 solution and centrifuged at 100 g for 2 min. The pelleted protoplasts were suspended in 100 μL of W5 solution and kept at 22^o^C for about 16 h in darkness. The fluorescence was observed by a confocal laser-scanning microscope using a Zeiss LSM 510 (Carl Zeiss, Jena, Germany).

**Table S1** Contents of L-theanine and its precursors in different tea tissues

| **μg/g F.W.** | **Root** | **Young leaf** | **Stem** | **Flower** |
| --- | --- | --- | --- | --- |
| L-Theanine | 4630.91 ±364.37  a | 2307.73 ±354.26  b | 1781.28 ±486.38  b | 534.72 ±159.46  c |
| L-Glutamate | 59.68 ±25.67  a | 262.07 ±63.32  b | 113.50 ±67.48  b | 657.45 ±277.89  b |
| Ethylamine | 420.44 ±100.98  a | 31.69 ±3.72  b | 56.63 ±1.09  b | 7.96 ±0.27  b |

Note: F.W., fresh weight. Different letters below data indicate significant differences by Duncan’s tests (*p* ≤ 0.05). Tissues (flower, young leaf, stem, and root) were collected from four-year-old tea seedlings of cv. Jinxuan in November, 2019.

**Table S2** Primers for vectors for transient overexpression in tobacco

| **Genes** |  | **Forward primers** | **Reverse primers** |
| --- | --- | --- | --- |
| *CsGS1.1* | 1^st^ | GGAGTGGGTGTTTTTGATCGATC | CCAAAACCCACAACTGCAAATAC |
|  | 2^nd^ | CGGTACCCGGGGATCCATGGCTCAGCTTTCAGATCTCA | CCATGGTGGCACTAGTTGGTTTCCACAGGATGGTGGT |
| *CsGS1.2* | 1^st^ | ATCGTGTGGTTTGTTTGGAGTGG | AACCCACAACTGCAAATAC |
|  | 2^nd^ | CGGTACCCGGGGATCCATGTCTTTGCTATCAGATCTCATCA | CCATGGTGGCACTAGTTGGCTTCCACAGCAGAGT |
| *CsGS1.3* | 1^st^ | GGGAGAGAGAACCAGAGCA | TACTATGAAAGAGCAGGGCAC |
|  | 2^nd^ | CGGTACCCGGGGATCCATGTCTCTTCTTTCCGATCTTTGCA | CCATGGTGGCACTAGTCGGTTTCCAGAGGATGGTGGT |
| *CsGS2* | 1^st^ | TCCGGCAGTCTCAGGGAG | CTCTAATATGCTTCTAAG |
|  | 2^nd^ | CGGTACCCGGGGATCCATGGCACAGATTTTGGCTCCTT | CCATGGTGGCACTAGTGACATTCATTGCCAGTTTCTGAGC |
| *CsTSΙ* | 1^st^ | GAGGGATAGAGTTTTGATCG | CTATAACCATTATCACATCTTC |
|  | 2^nd^ | CGGTACCCGGGGATCCATGGAGAAATTTGCAGAGCTGA | CCATGGTGGCACTAGTATAGCGATGTATAAGTTGCTTGT |

**Table S3** Primers for qRT-PCR analyses

| Genes | Forward primers | Reverse primers | GenBank ID |
| --- | --- | --- | --- |
| *CsGS1.1* | CAGCACCAAGTCTACGAGGA | AATCATGGAAGTAACCACA | MG778703 |
| *CsGS1.2* | TGCAAACCGTGGTGCATCTG | GTTTCCACAGGATGGTGGTAG | MG778705 |
| *CsGS1.3* | TGGCCGATTGGTTGGCCTG | ACAACTGATCTCCAGAACT | MG778704 |
| *CsGS2* | CTGGAACGGTGCAGGATGC | GCCCCACGCGGATTGAACA | MG778706 |
| *CsTSΙ* | GTTGATGTTTCTGGGCAGCA | CTCACCCACACCAGTCAGAT | TEA015198.1 |
| *Csβ-actin* | GCCATATTTGATTGGAATGG | GGTGCCACAACCTTGATCTT | HQ420251.1 |

**Table S4** Primers for CsGS/TS-YFP-vectors in *Arabidopsis* protoplasts

| **Genes** | **Forward primers** | **Reverse primers** |
| --- | --- | --- |
| *CsGS1.1* | GAATTCTGCAGTCGAATGGCTCAGCTTTCAGATCTCATCA | CTCACCATCAGGATCTTTGGTTTCCACAGGATGGTGGT |
| *CsGS1.2* | GAATTCTGCAGTCGAATGTCTTTGCTATCAGATCTCATCA | CTCACCATCAGGATCTTTGGCTTCCACAGCAGAG |
| *CsGS1.3* | GAATTCTGCAGTCGAATGTCTCTTCTTTCCGATCTTTGC | CTCACCATCAGGATCTTCGGTTTCCAGAGGATGGTGG |
| *CsGS2* | GAATTCTGCAGTCGAATGGCACAGATTTTGGCTCCTTC | CTCACCATCAGGATCTTGACATTCATTGCCAGTTTCTGAG |
| *CsGS2*  （without its predicted N-terminal chloroplast transit peptide） | GAATTCTGCAGTCGAATGGTGTTTGCTCTACAGTCCG | CTCACCATCAGGATCTTGACATTCATTGCCAGTTTCTGAG |
| *CsTSΙ* | GAATTCTGCAGTCGAATGGAGAAATTTGCAGAGCTGAG | CTCACCATCAGGATCGGATAGCGATGTATAAGTTGCTTG |

**Table S5** Primers for YFP-CsGS/TS vectors in *Arabidopsis* protoplasts

| **Genes** | **Forward primers** | **Reverse primers** |
| --- | --- | --- |
| *CsGS1.1* | GACGAGCTGTACAAGATGGCTCAGCTTTCAGATCTCATC | CTAGGTGGATCTTCATCATGGTTTCCACAGGATGGTGG |
| *CsGS1.2* | GACGAGCTGTACAAGATGTCTTTGCTATCAGATCTCATC | CTAGGTGGATCTTCATCATGGCTTCCACAGCAG |
| *CsGS1.3* | GACGAGCTGTACAAGATGTCTCTTCTTTCCGATCTTTGC | CTAGGTGGATCTTCATTACGGTTTCCAGAGGATGGTGG |
| *CsGS2* | GACGAGCTGTACAAGATGGCACAGATTTTGGCTCCTTC | CTAGGTGGATCTTCATTAGACATTCATTGCCAGTTTCTGA |
| *CsTSΙ* | GACGAGCTGTACAAGATGGAGAAATTTGCAGAGCTGAG | CTAGGTGGATCTTCATCAATAGCGATGTATAAGTTGCTTG |
| *pSAT6-EYFP-N1* | TGAAGATCCACCTAGTCTAGAGTCC | CTTGTACAGCTCGTCCATGCC |


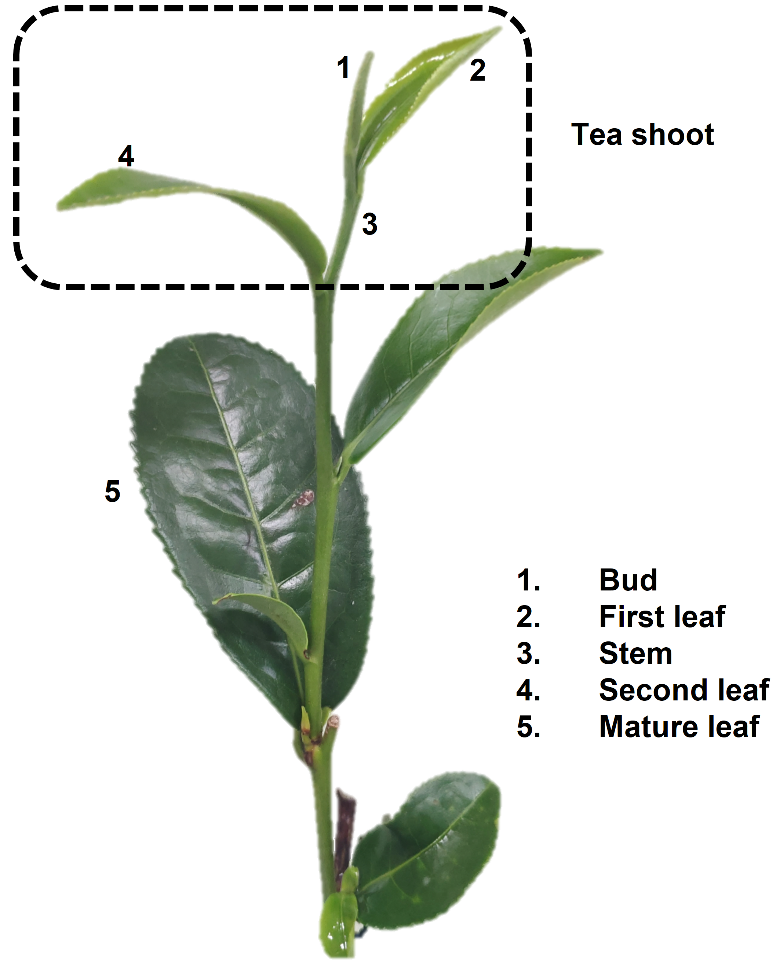


**Figure S1 The photo of tea shoot tissue.**

Tea shoot tissue containing bud, first leaf, stem and second leaf were picked to study the L-theanine accumulation mechanism under shade environment.

**
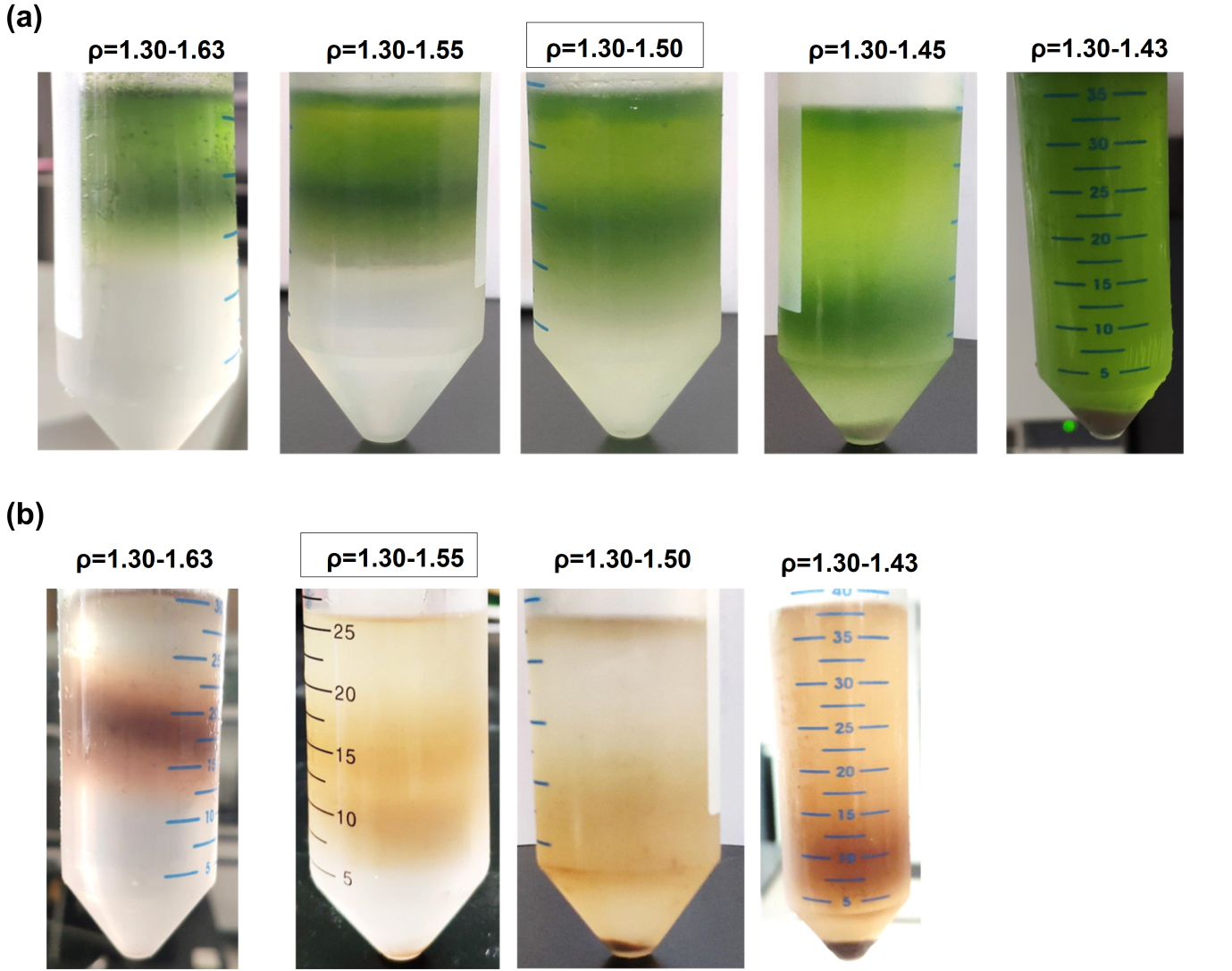
**

**Figure S2 Optimization of the density for separation of fractions from tea tissues using the nonaqueous fractionation method.**

(a) Tea shoot tissue and (b) root tissue. The density was optimized to separate the fractions in tea plant shoot and root tissues, a smaller density range was used of 1.30 to 1.50 g/mL for tea shoot material and 1.30 to 1.55 g/mL for tea root material. The optimal densities for shoot and root, respectively, are boxed. The tissues of tea shoot and root were collected from twenties-year-old tea plants (cv. Jinxuan) in March, 2019.

**
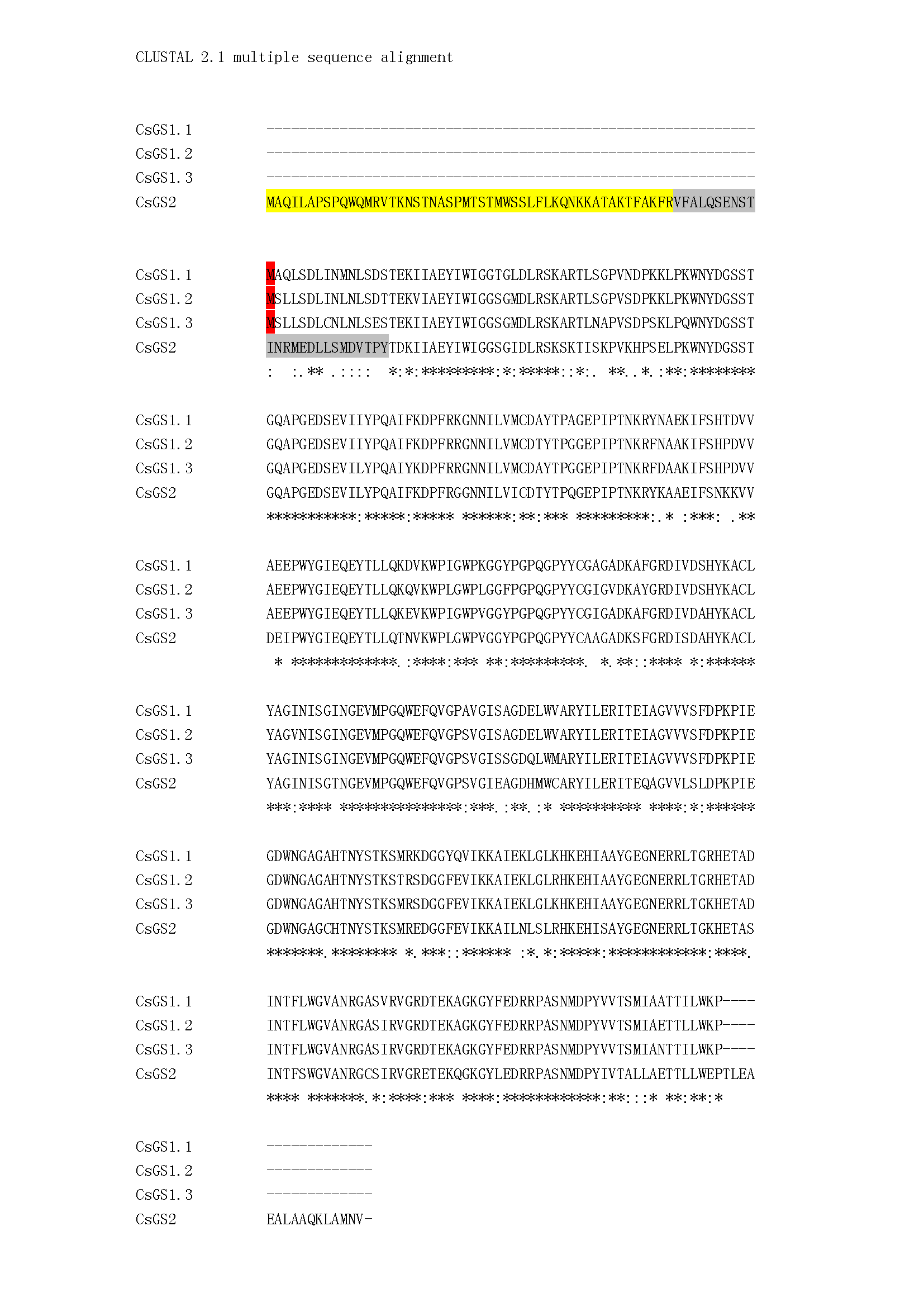
**

**Figure S3 CsGSs amino acid sequences alignment by Clustal.**

The amino acid sequences highlighted in yellow were predicted as a chloroplast transit peptide. The amino acid sequences highlighted in grey were predicted as mitochondria. The amino acid sequence highlighted in red was the start codon of the protein sequence of CsGS1.1, CsGS1.2, and CsGS1.3.

**
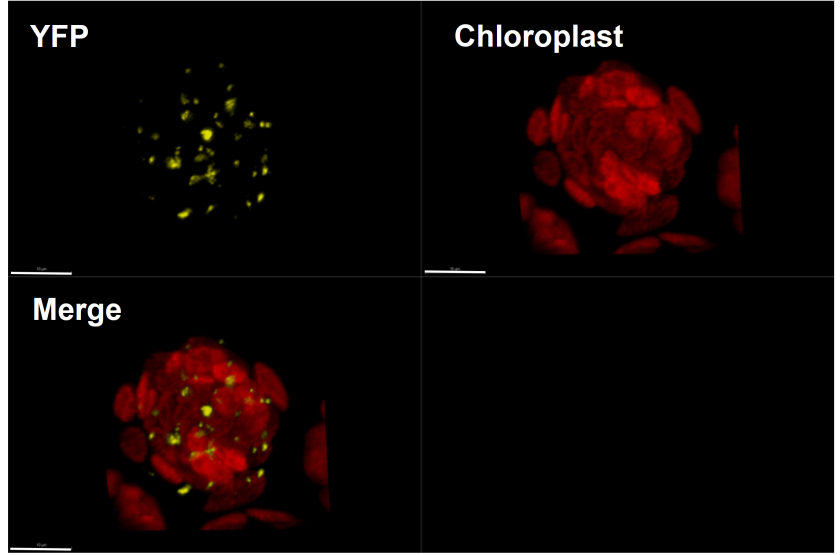
**

**Figure S4 Subcellular localization for CsGS2-YFP without its predict N-terminal chloroplast transit peptide.**

Bar = 10 μm.

**
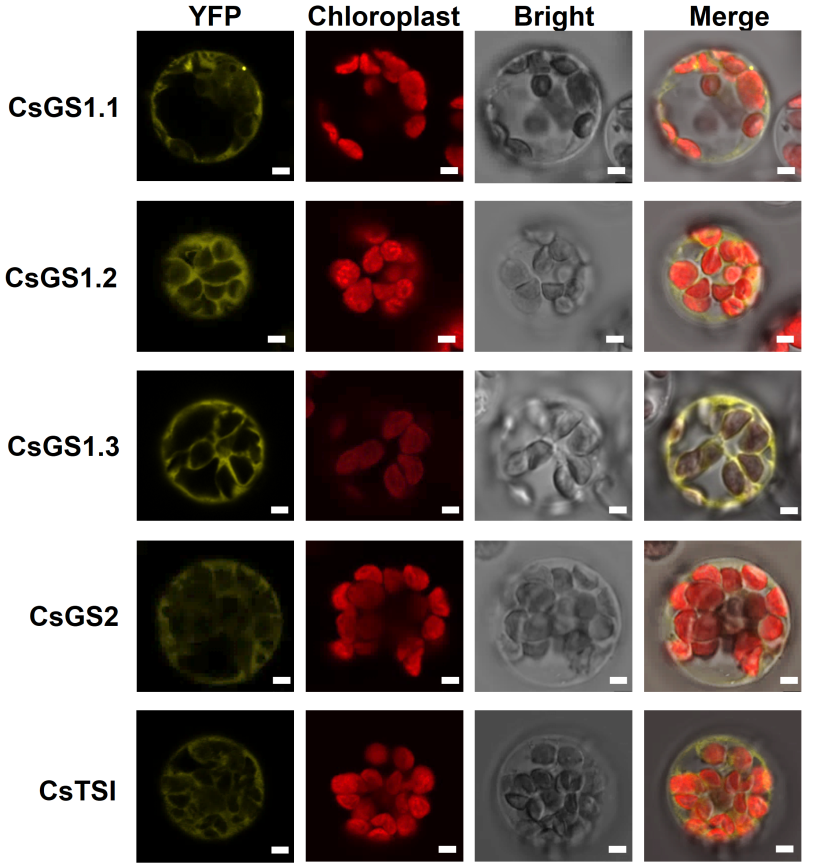
**

**Figure S5 Subcellular localizations for YFP-CsGS/TS.**

Bar = 5 μm.

**
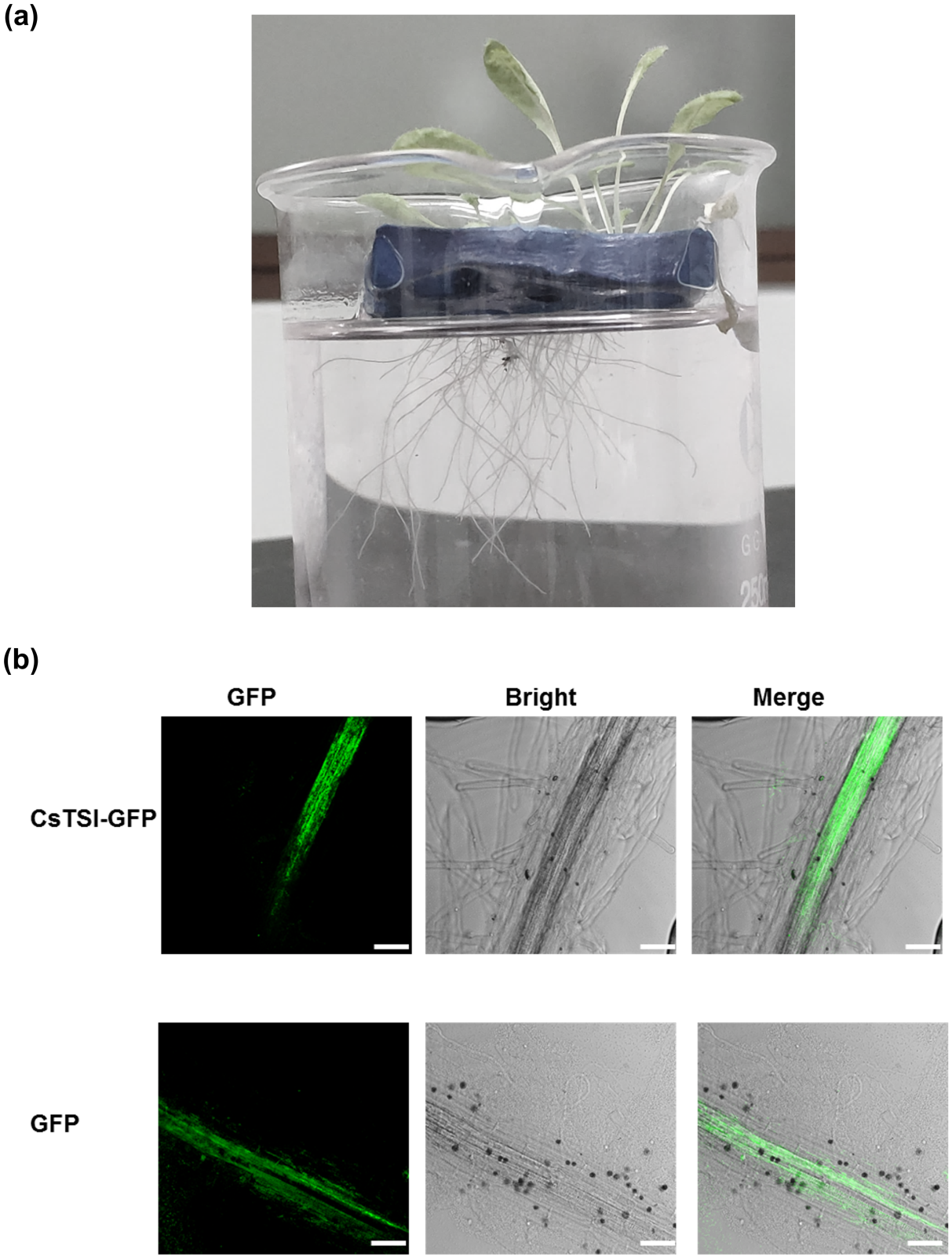
**

**Figure S6 *Arabidopsis* hydroponics root culture (a) and subcellular localizations of CsTSІ-GFP (b).**

Bar = 50 μm.

**
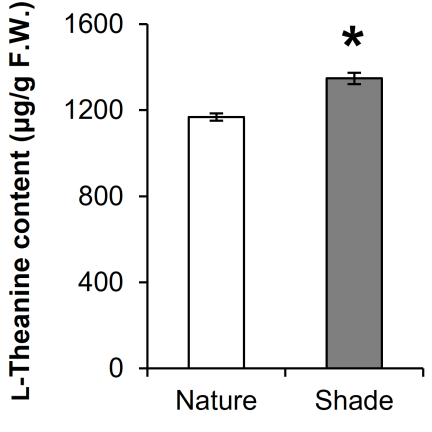
**

**Figure S7 Effect of shade treatment on L-theanine content in tea shoot tissue.**

Tea shoots from twenties year old tea plants (cv. Jinxuan) were used and the shade treatment experiment was conducted for two weeks from the 5^th^, November 2019 to the 19^th^, November 2019. Data show mRNA levels relative to actin mRNA and are expressed as means ± SD (n=3). * *p* ≤0.05 using Student’s *t* test.

**
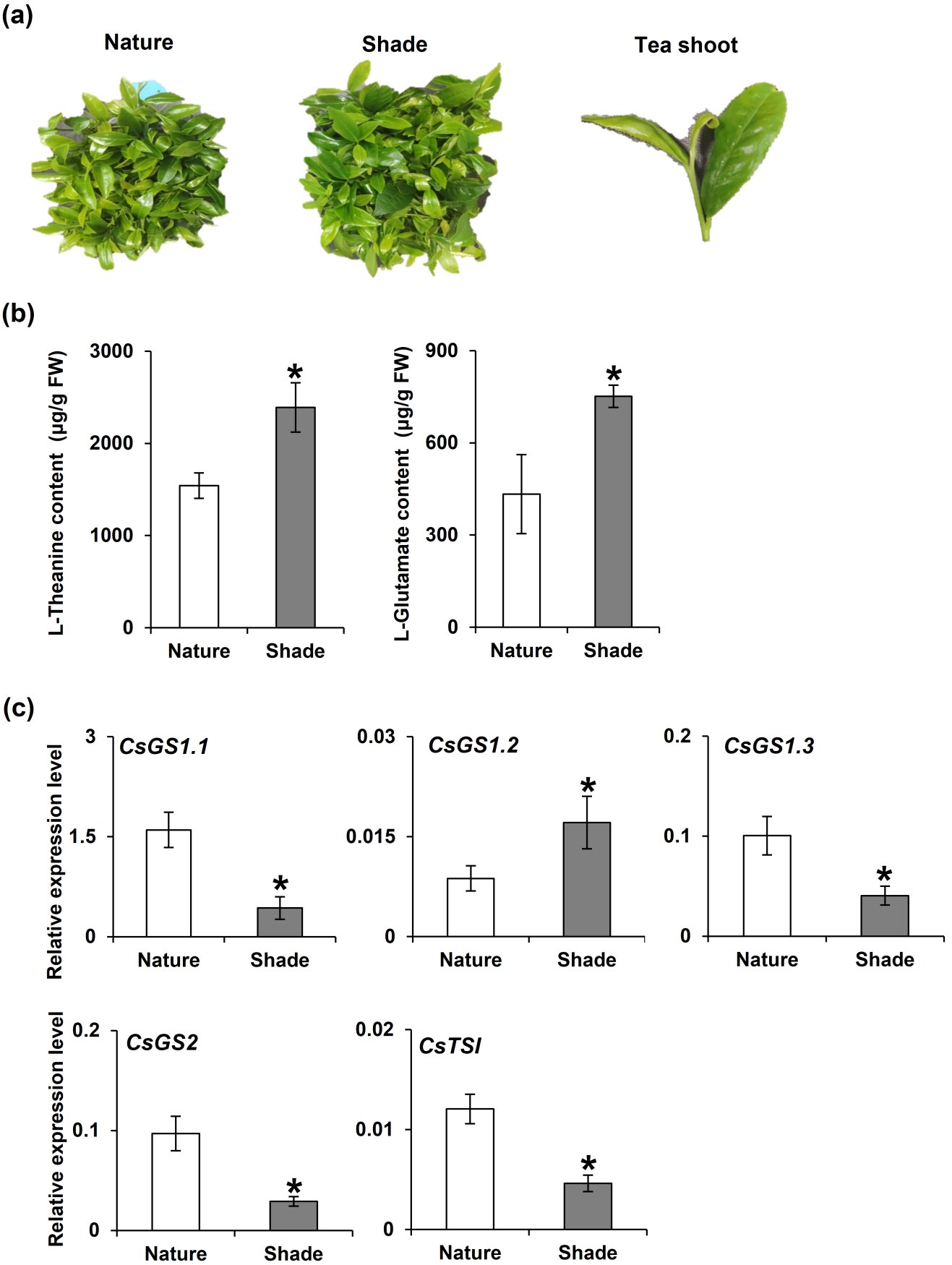
**

**Figure S8 Effects of shade treatment on L-theanine accumulation in tea shoot tissue.**

(a) Photographs showing the twenties-year-old tea plant (cv. Jinxuan) grown in natural day/night cycles and long-term shading environment for two weeks from the 24^th^, April 2020 to the 8^th^, May 2020.

(b) Comparisons of L-theanine and L-glutamate contents in the shoot of tea grown in natural light/dark cycles and long-term shading environment for two weeks.

(c) mRNA levels of *CsGS1.1*, *CsGS1.2*, *CsGS1.3*, *CsGS2* and *CsTSΙ* in the shoot of tea grown in natural light/dark cycles and long-term shading environment for two weeks. Data show mRNA levels relative to actin mRNA and are expressed as means ± SD (n=3). * *p* ≤0.05 using Student’s *t* test.

**
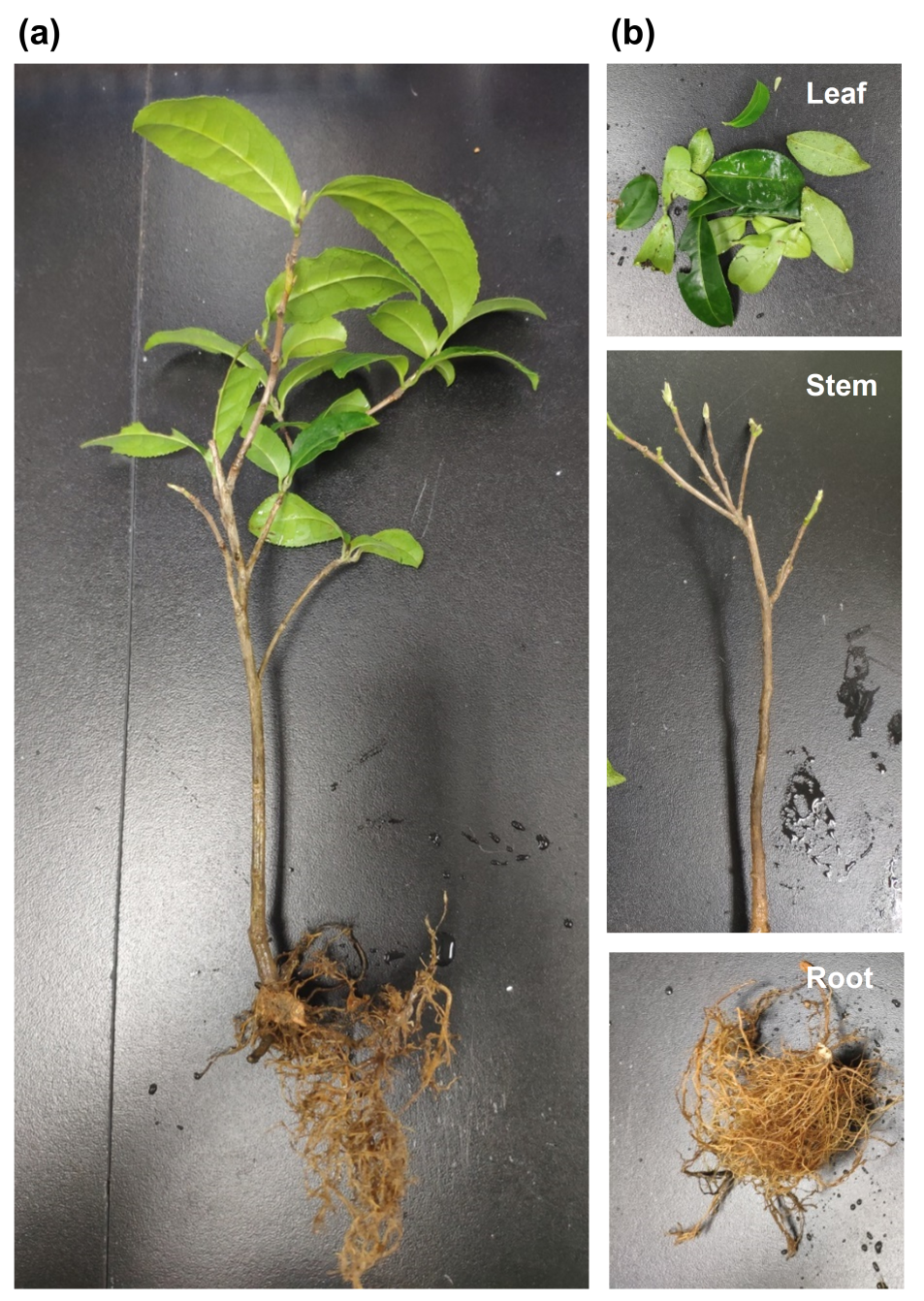
**

**Figure S9 Two-year-old tea seedling used for studying L-theanine accumulation mechanism under the shade treatment.**

The shade treatment time was from the 24^th^, April 2020 to the 8^th^, May 2020. (a) The whole two-year-old tea seedling plant (cv. Jinxuan). (b) Leaf, stem, and root tissues were separated to study the L-theanine accumulation mechanism.

**
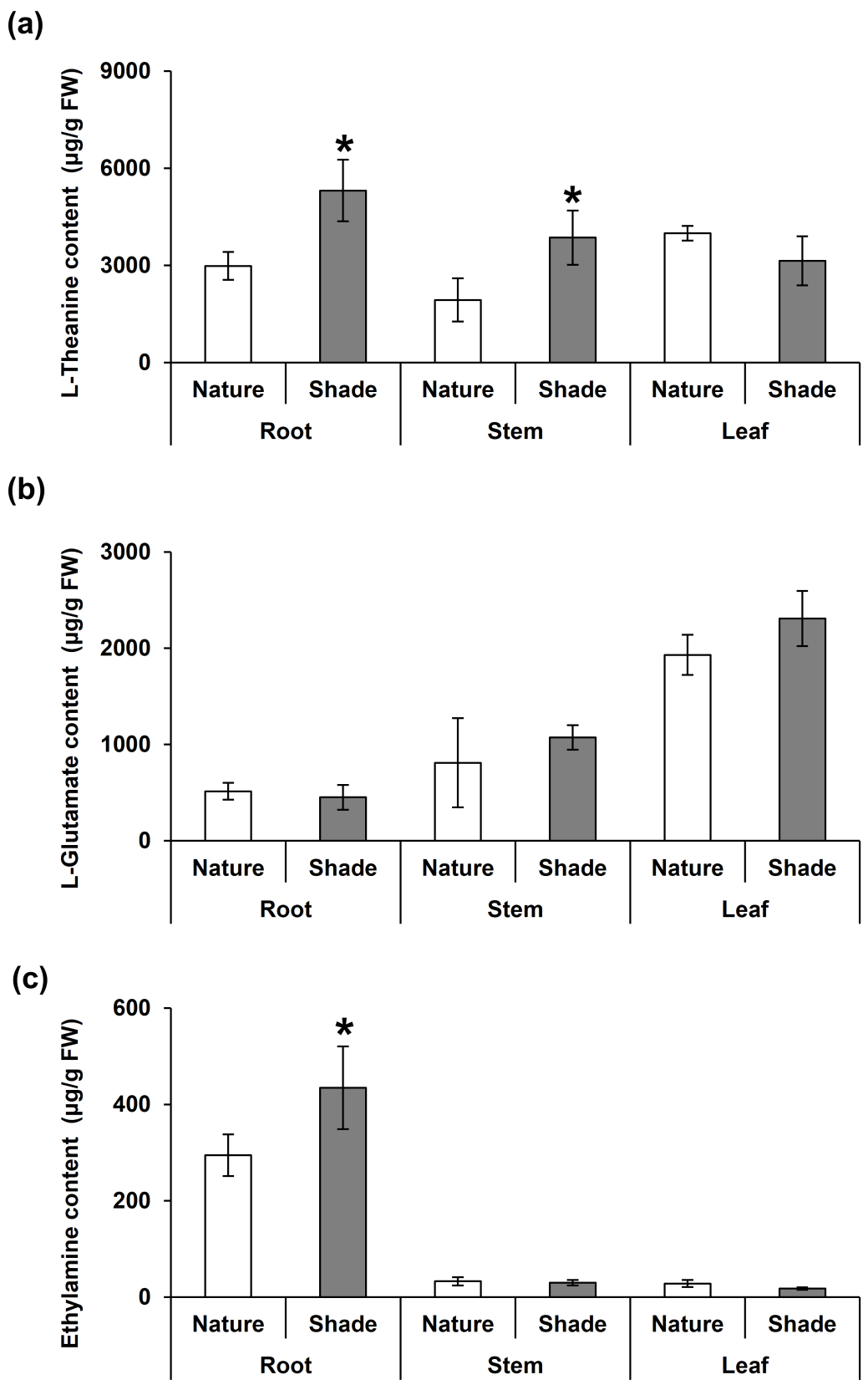
**

**Figure S10** **Analyses of L-theanine (a) and its precursors of L-glutamate (b) and ethylamine (c) contents in two-year-old tea seedling after long-term shading treatment.**

The shade treatment time on the two-year-old tea seedling (cv. Jinxuan) was from the 24^th^, April 2020 to the 8^th^, May 2020. * *p* ≤0.05 using Student’s *t* test.

**
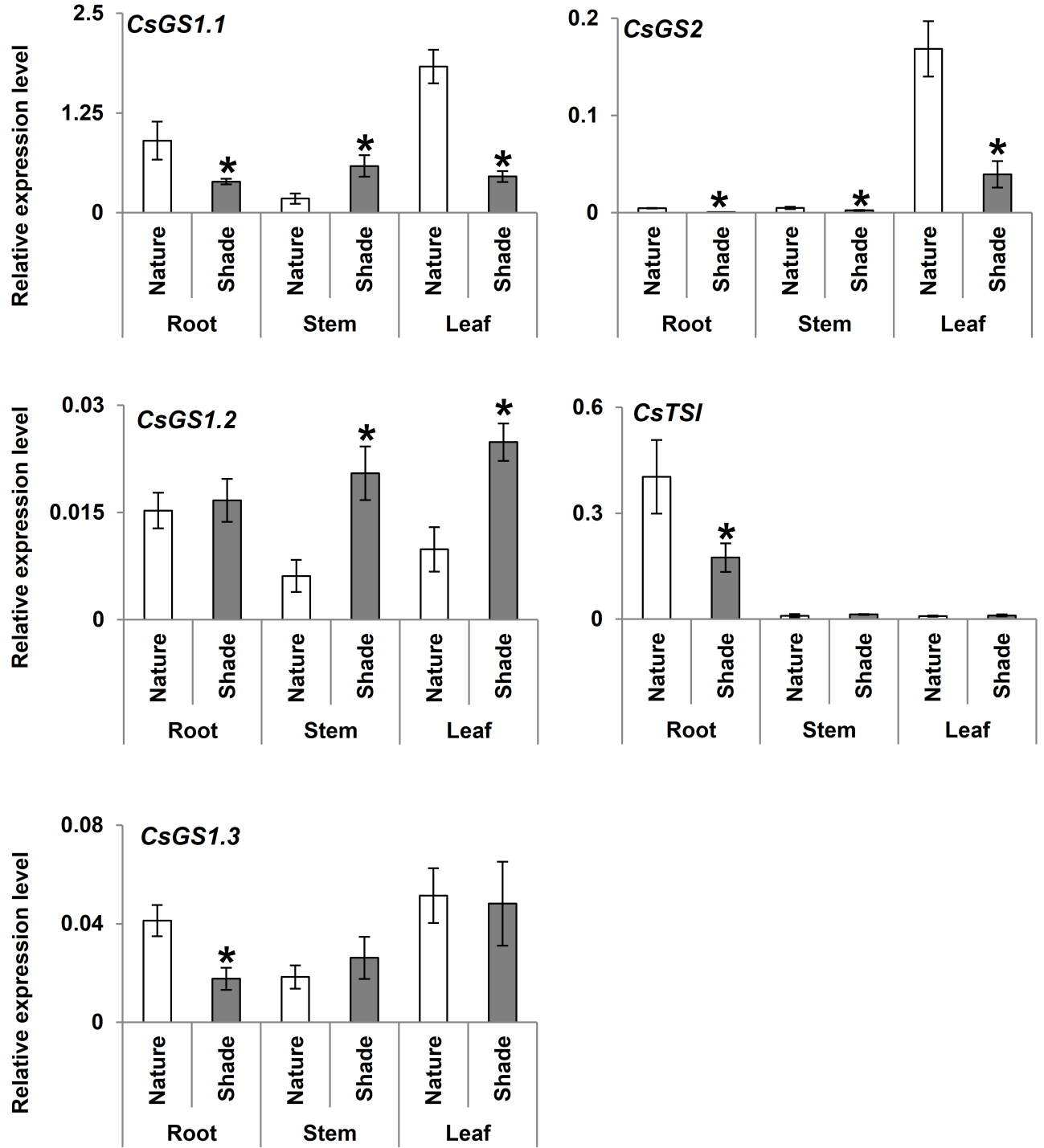
**

**Figure S11 Analyses of mRNA levels of *CsGS1.1*, *CsGS1.2*, *CsGS1.3*, *CsGS2*, and *CsTSΙ* in the tissues of two-year-old tea seedling after long-term shading treatment.**

The shade treatment time on the two-year-old tea seedling (cv. Jinxuan) was from the 24^th^, April 2020 to the 8^th^, May 2020. Data show mRNA levels relative to actin mRNA and are expressed as means ± SD (n=3). * *p* ≤0.05 using Student’s *t* test.

**References**

Almeida, C., Fernandes, J.O. and Cunha, S.C. (2012) A novel dispersive liquid–liquid microextraction (DLLME) gas chromatography-mass spectrometry (GC–MS) method for the determination of eighteen biogenic amines in beer. *Food Control*. **25**, 380-388.

Cheng, S.H., Fu, X.M., Wang, X.Q., Liao, Y.Y., Zeng, L.T., Dong, F. and Yang, Z.Y. (2017) Studies on the biochemical formation pathway of the amino acid l-theanine in tea (*Camellia sinensis*) and other plants. *J. Agric. Food Chem.* **65**, 7210-7216.

Farré, E.M., Tiessen, A., Roessner, U., Geigenberger, P., Trethewey, R.N. and Willmitzer, L. (2001) Analysis of the compartmentation of glycolytic intermediates, nucleotides, sugars, organic acids, amino acids, and sugar alcohols in potato tubers using a nonaqueous fractionation method. *Plant Physiol.* **127**, 685-700.

Krueger, S., Steinhauser, D., Lisec, J. and Giavalisco, P. (2014) Analysis of subcellular metabolite distributions within *arabidopsis thaliana* leaf tissue: a primer for subcellular metabolomics. In: *Arabidopsis Protocols* (Sanchez-Serrano, J.J. and Salinas, J. eds), pp. 575-596. Totowa, NJ: Humana Press, 575-596.

Stitt, M., Lilley, R.M., Gerhardt, R. and Heldt, H.W. (1989) Metabolite levels in specific cells and subcellular compartments of plant leaves. In: *Methods Enzymol.* pp. 518-552.

Zhou, Y., Zeng, L., Liu, X., Gui, J., Mei, X., Fu, X., Dong, F., Tang, J., Zhang, L. and Yang, Z. (2017) Formation of (*E*)-nerolidol in tea (*Camellia sinensis*) leaves exposed to multiple stresses during tea manufacturing. *Food Chem.* **231**, 78-86.
